# Variability and repeatability of spinal manipulation force–time characteristics in thoracic spinal manipulation on a manikin

**DOI:** 10.1186/s12998-024-00551-2

**Published:** 2024-11-11

**Authors:** Luana Nyirö, Lindsay M. Gorrell, Valentina Cecchini, Carlo Menon, Mohamed Elgendi, Petra Schweinhardt

**Affiliations:** 1 Department of Chiropractic Medicine, Integrative Spinal Research, Balgrist University Hospital, University of Zurich, Zurich, Switzerland; 2 Biomedical and Mobile Health Technology Lab, Department of Health Sciences and Technology, ETH Zurich, Zurich, Switzerland; 3 Department of Biomedical Engineering and Biotechnology, Khalifa University of Science and Technology, Abu Dhabi, United Arab Emirates; 4 Healthcare Engineering Innovation Group , Khalifa University of Science and Technology, Abu Dhabi, United Arab Emirates

**Keywords:** Spinal manipulation, Biomechanics, Force–time characteristics, Chiropractic, Spine Pain

## Abstract

**Background:**

As part of multimodal therapy, spinal manipulation (SM) is a recommended and effective treatment for musculoskeletal pain. However, the underlying physiological mechanisms for pain relief are largely unknown. SM thrusts can be described and quantified using force–time characteristics (e.g. preload force, peak force, thrust speed, thrust duration, and thrust impulse). If these biomechanical parameters of SM are important for clinical outcomes, a large variability in the delivery of SM could lead to inconsistent responses and could thereby potentially mask a significant clinical effect. Our goal was to determine variability, and repeatability of thoracic spinal manipulation (SM) force–time profiles in a sample of Swiss chiropractors.

**Methods:**

All interventions were performed on a human analogue manikin. Participating chiropractors received three case scenarios with the following scenarios: 50-year-old male patient, 30-year-old male athlete, and a 70-year-old female patient, each presenting with uncomplicated musculoskeletal thoracic pain. Clinicians were asked to perform three consecutive thoracic SM thrusts for each of the scenarios and repeated the same interventions after 24–48 h.

**Results:**

Eighty-one chiropractors participated in the study, including 32 females (39.5%) with a mean age of 45.22 ± 12.96 years. The variability in SM force–time characteristics between clinicians was substantial, with preload forces ranging from 4.50 to 450.25 N and peak forces ranging from 146.08 to 1285.17 N. Significant differences between case scenarios were observed for peak force (*p* < 0.0001), maximum thrust speed (*p* = 0.0002), and thrust impulse (*p* = 0.0004). Except for thrust duration, repeatability within and between sessions was fair to excellent (ICCs between 0.578 and 0.957).

**Conclusion:**

Substantial variability in application of SM was evident across clinicians and between case scenarios. Despite substantial clinician-dependent variability, the high repeatability of thoracic SM thrusts suggests a level of standardization in SM delivery, indicating that chiropractors might have ‘their’ individual force–time profile that they are capable to reproduce. Further research based on these findings should explore how to enhance the consistency, effectiveness, and safety of thoracic SM delivered clinically to humans.

**Supplementary Information:**

The online version contains supplementary material available at 10.1186/s12998-024-00551-2.

## Background

Musculoskeletal spinal pain is among the main causes of years lived with disability worldwide [1–4]. Spinal manipulation (SM) therapy has long been a component of manual therapy and physical rehabilitation programs for spinal pain and is practiced by a wide variety of clinicians including physical therapists, physicians with manual medicine training, and chiropractors. Recent comprehensive reviews suggest that SM is effective for the treatment of acute and chronic musculoskeletal pain [5–8]. It is a recommended treatment for musculoskeletal disorders as part of multimodal therapy, especially as a modality for pain relief [9, 10].

SM is considered a complex motor skill, mastered with comprehensive training [11, 12]. It is characterized by a single, high-velocity, low-amplitude (HVLA) thrust to a joint. The maneuver aims to move the joint beyond its usual physiological range of motion, while staying within its anatomical limits [13]. Therefore, the use of SM techniques relies on the skill of the clinician and their perception of the nature and degree of joint movement [12]. Much attention is paid to learning the correct thrust technique, as this is anecdotally considered important for clinical outcomes [11, 14]. However, the underlying physiological mechanisms for manipulation-related pain relief remain largely unknown [15]. Although this does not negate the clinical effects of SM, it hinders acceptance of the procedure by the wider scientific and healthcare communities and restrains development of rational strategies for improving the delivery of SM.

As muscle spindle afferents, Golgi tendon organ afferents and small-diameter sensory nerve fibers are stimulated by SM, it has been suggested that a mechanical impetus is necessary to initiate a chain of neurophysiological responses [16, 17]. However, evaluating SM delivery is a complex task which is complicated by a paucity of validated and objective measures [18, 19]. As an approximate approach, SM thrusts can be described and quantified using force–time parameters such as preload and peak force, thrust duration and rate of force application [20–24]. In mechanistic studies on SM, physiological and biomechanical effects are more and more often analyzed and interpreted in relation to their SM parameters, with increasing evidence for the existence of a dose–response relationship [21, 25–31]. Nonetheless, these responses are predominantly transient and there is so far no established association with meaningful change in clinical outcomes (e.g. decreased pain and increased range of motion) [21, 32]. Quantifying the force–time characteristics represents the first step towards identifying potential active components responsible for clinical efficacy of the intervention.

Despite these considerations, little attention has so far been paid to the systematic recording of SM parameters. If force–time characteristics are important for clinical outcomes, a large variability in the delivery of SM could lead to inconsistent responses and could thereby potentially mask a significant clinical effect [33].

Since SM treatments are usually provided over a treatment period consisting of several consultations, repeatability of the intervention is another important factor for facilitating reproducible research findings, ensuring reliable and accurate treatment outcomes, validating the efficacy of the intervention, and enhancing patient safety [34]. Still, there is a lack of studies evaluating the repeatability of SM [18]. Even less research is done on variability of SM delivery between clinicians [35, 36]. Therefore, the objective of this study was to assess the variability and repeatability of SM force–time characteristics among chiropractors, and to explore potential factors influencing such variability and repeatability.

## Methods

### Study setting and participants

All chiropractors licensed in Switzerland were eligible for enrolment in the trial. Data collection was performed during the 3-day annual Swiss chiropractic congress, held from September 1st to 3rd, 2022, in Lugano, Switzerland. All members of the Swiss Chiropractic Association were informed about the data collection via flyer and oral information during the conference opening. Interested clinicians self-enrolled and received a personal study ID which they kept throughout the whole study. Data collection was performed in a quiet room at the conference venue.

Data collection of health-related and demographic information was anonymous and no information or health-related data were collected in this study. A declaration of non-responsibility was received from the local ethics board ‘Kantonale Ethikkomission Zürich, KEK’ (BASEC-Nr. Req 2020-00932).

### Baseline characteristics

All participants answered a questionnaire on a tablet prior to data collection. Baseline information included age, sex, clinical experience, weight, height, country of education, and preferred manipulation techniques of the thoracic spine. Additionally, grip strength was measured with a calibrated dynamometer by trained study assistants using standardized instructions.

### SM interventions

**Fig. 1:**
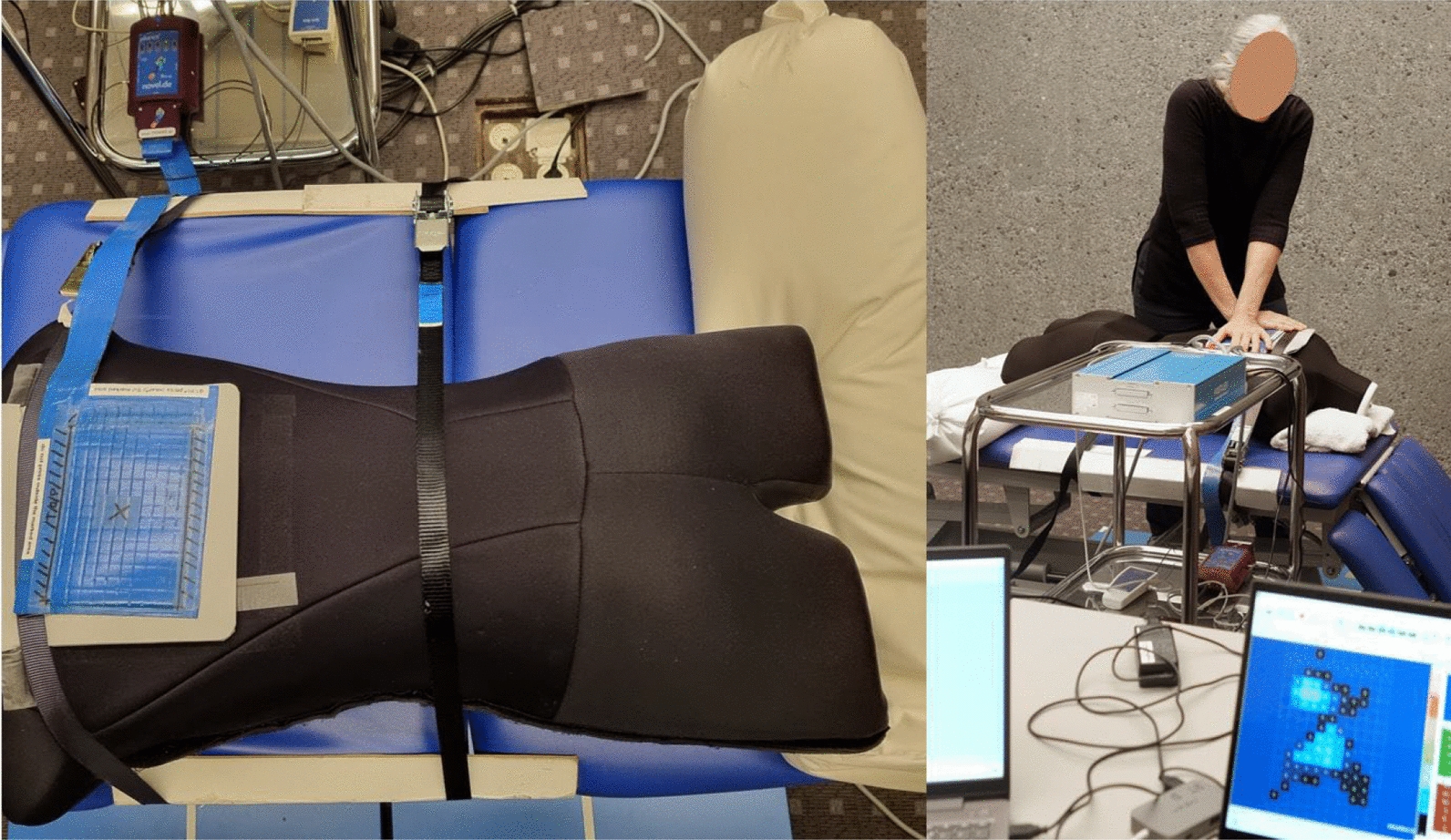
Study set-up. (Left image) The manikin was fixed to the treatment table using a clamping set. The sensor mat (blue) was positioned over the target vertebra (cross marking) by the study personnel, and the sensor area was marked with a black border; (Right image) A chiropractor performing the SM intervention while the screen displays the pressure sensed by the sensor grid

All SM interventions were performed on a human-analogue manikin (HAM™, Canadian Memorial Chiropractic College, Toronto, Canada) using a standardized protocol. The SM intervention consisted of a HVLA mid-thoracic SM, with the manikin in prone position and the treatment force directed posterior to anterior [13, 37, 38]. The contact area and the position of the segment to be treated was defined by the study set-up (approximately Th4—Th5, in the center of the sensor area, see Fig. 1).

Immediately before data collection, all participants underwent a familiarization session where they oriented themselves with the set-up (e.g. feet positioning, touching and palpation of the manikin) and performed five SM trial runs with the exact replication of the set-up used for data collection. Participants were asked to position themselves as comfortable as possible, and as similarly as they would do in their own practice, to adjust the table height if needed, and to keep their main contact within the marked sensor area. No further instructions were given regarding the SM technique or choice of contact, but rather this was left to the practitioner’s discretion. Familiarization was supervised by trained study assistants using standardized instructions.

**Fig. 2:**
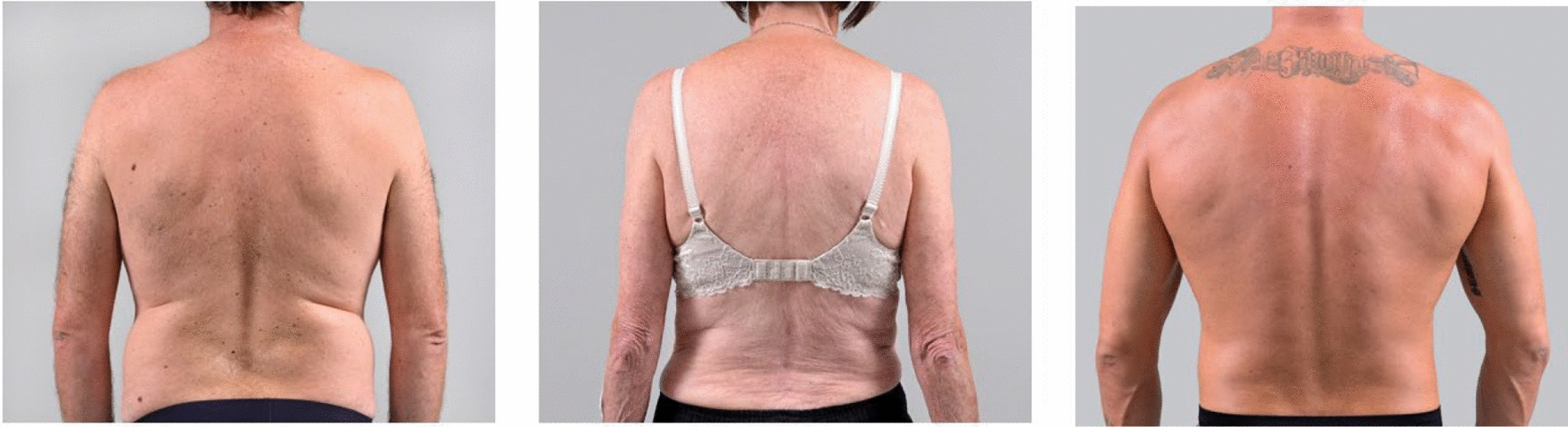
Case scenarios for spinal manipulation interventions. (Left image) Scenario 1:”standard” 50-year-old healthy male patient (used as a reference scenario); Middle image) Scenario 2: 70-year-old healthy female patient; (Right image) Scenario 3: 30-year-old healthy male athlete

During data collection, the participants received three different case scenarios with an image of the corresponding patient’s back, describing the following scenarios:

All scenarios were described as a returning patient for sporadic musculoskeletal mid-thoracic pain, without any red flags, and previously responding well to mid-thoracic SM (see Fig. 2). The exact scenarios and provided clinical information can be found in the supplementary file 1. No further criteria (e.g. specifications regarding restricted movement directions) were given to the clinicians.

Following the review of all three scenarios, participants were instructed to perform three SM interventions on the manikin for each scenario, with the corresponding scenario being presented in front of the clinician. The thrusts were recorded consecutively, but in separate measurement files, multiple thrusts per recording were not allowed.

**Fig. 3:**
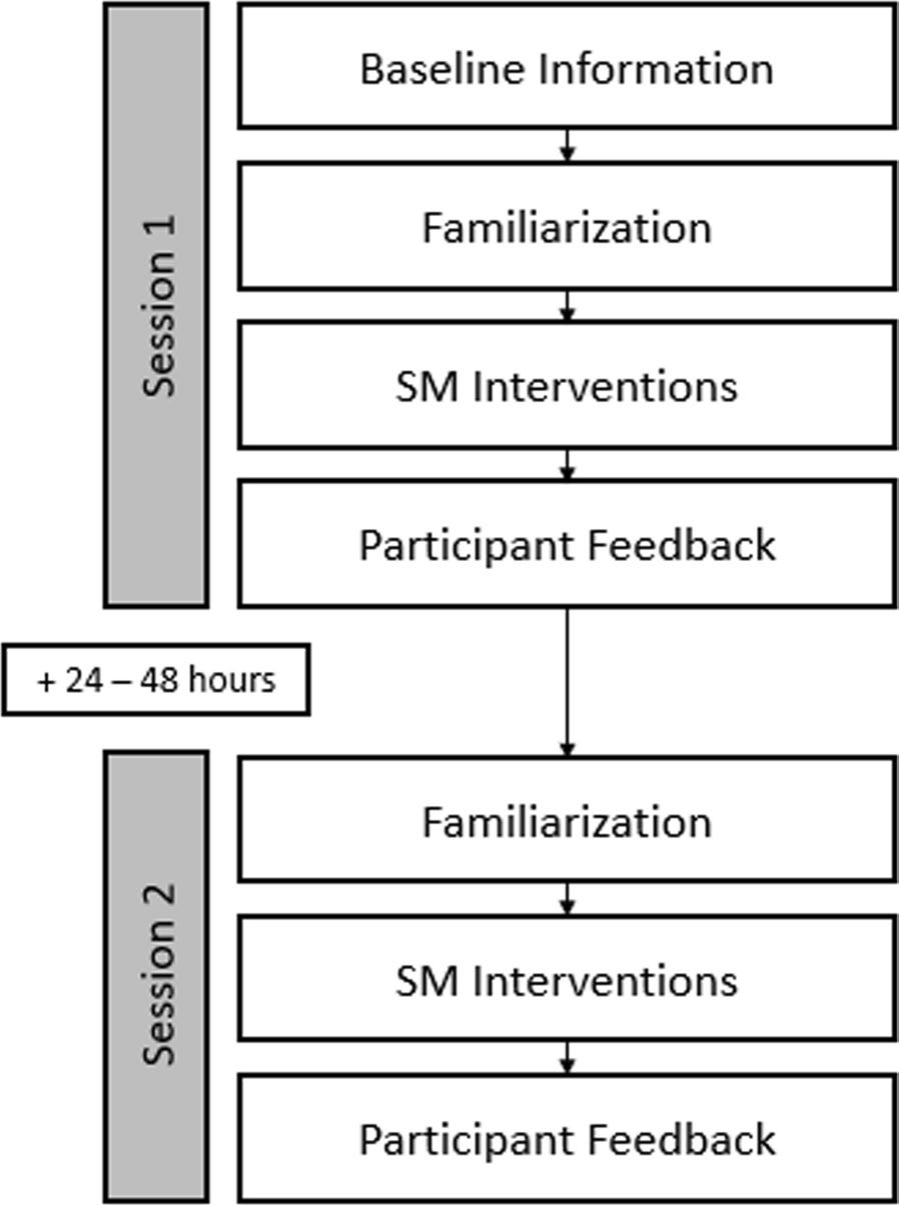
Study Timeline. The study consisted of two sessions. Session 1 included collection of baseline information, familiarization, SM interventions, and participant feedback. After a 24–48 h interval, familiarization, SM interventions and participant feedback were repeated during Session 2

First, all participants performed three thrusts for Scenario 1. The order of scenario 2 and 3 was randomized between participants using a balanced randomization sequence generated by a random number generator. Regarding positioning of hands, feet, and table for the SM interventions, the same instructions were given as for the familiarization session. Additionally, participants were instructed to visualize the patient corresponding to the described scenario while executing the thrusts, to perform the three thrusts for a given scenario as similarly as possible, and to perform the thrusts as closely as possible to their typical clinical routine. The supervising study investigator (LN) recorded the contact used (hand position) for each thrust (Fig. 3).

Between 24 and 48 h later, participants returned for a second study visit and were requested to again deliver SM to all three patient scenarios using the same treatment force employed during the initial visit. All interventions were monitored by the same two study investigators (LN, LG) in the same roles. After the intervention, participants completed a survey to rate the comparability of the intervention to their everyday clinical practice and to provide individual feedback or comments.

### Measurement device

Force–time profiles were measured using a flexible force-sensing system (Novel pliance®-xf-16 system, Munich, Germany; analyzer PXF440, software Novel Database 28.3.8.9). Sensor matrix was 11 × 16 sensors (1 sensor/cm^2^) with the sensor calibrated up to a peak load of 1.2 MPa, and a sampling rate of 100 Hz.

Measurement error of the system for static loads was reported to be within a maximum 5% error by the manufacturer and pilot tests in our laboratory prior to data collection with a force plate (Kistler®, Winterthur, Switzerland; type 9260AA6, sampling rate 1000 Hz) served as a reference standard and confirmed a measurement error below 5% during dynamic thrusts.

### Force–time characteristics

**Fig. 4:**
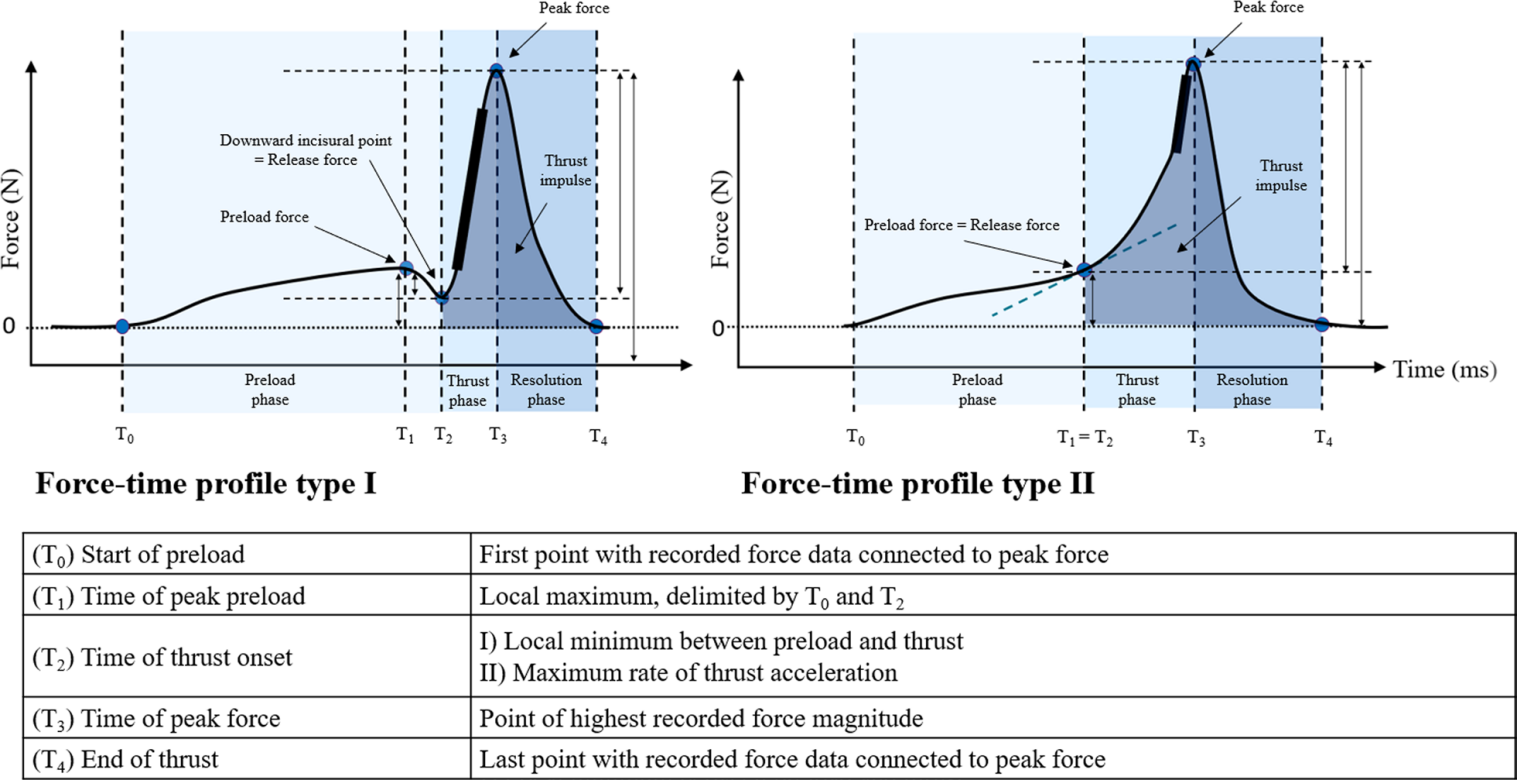
Force–time profiles of spinal manipulation

A custom-implemented graphical user interface written in MATLAB (MathWorks, Inc, Natick, MA) was used to extract the start of preload, time of peak preload, time of thrust onset, time of peak force and end of thrust as well as the time-corresponding forces (see Fig. 4). Based on these parameters, further relevant characteristics of the force–time profiles, i.e. maximum thrust speed, rate of force application, thrust impulse, and thrust duration, were calculated [39].

Two typical forms of force–time profiles were identified (see Fig. 4), with the predominant thrust strategy (thrust strategy I) including a local minimum, in the literature also referred to as a downward incisural point [20]. In a relevant proportion of thrusts (162 (13.1%) of recorded interventions), we observed a different force-development strategy (thrust strategy II), representing a monotonic transition from preload to thrust, which has not yet been reported in the literature. After discussion among the authors, the maximum rate of thrust acceleration was defined as thrust onset for these cases, in order to enable comparability between the interventions.

The accuracy of the automated extraction was verified by visual inspection, and corrected manually if necessary, by the main investigator (LN). The specific characteristics extracted were adapted from Downie et al. [20], Gorrell et al. [22] and Gyer et al. [21] and are summarized in Table 1.

**Table 1:** Definition of force–time profile characteristics

Force–time characteristic	Definition
Preload force, N	Local maximum, delimited by T_0_ and T_2_
Release force, N	Force at T_2_
Δ Preload and release force, N	Force at T_1_–Force at T_2_
Peak force, N	Force at T_3_
Thrust force, N	Force at T_3_–Force at T_2_
Maximum thrust speed, N/s	Maximum of first derivative of the force time-profile
Rate of force application, N/s	(Peak force–release force)/thrust duration
Thrust impulse, Ns	Area under the curve from T_2_ to T_4_
Preload duration, ms	T_2_–T_0_
Thrust duration, ms	T_3_–T_2_
Resolution duration, ms	T_4_–T_3_
Total duration, ms	T_4_–T_0_

N = Newton; N/s = Newtons per second; Ns = Newton second; ms = millisecond

### Statistical analysis

Statistical analyses were performed using the statistical software R version 4.2.2 and R Studio version 2023.12.0 + 369. A *p*-value < 0.05 was considered statistically significant. Except for within-session repeatability, the biomechanical characteristics of the three consecutive thrusts were averaged for each case scenario.

#### Participants

Baseline characteristics of participating chiropractors were summarized using descriptive statistics.

#### Variability

Characteristics of the force–time profiles were summarized using descriptive statistics. Variability across chiropractors was described using standard deviations, range, and interquartile range. To better understand whether certain characteristics are interdependent or whether they vary independently across individuals, correlations between the different force–time variables were calculated using Pearson correlation analysis. The differences between the three patient scenarios were investigated using a repeated measures Analysis of Variance (ANOVA) with post-hoc Tukey’s HSD tests. Partial eta-squared (η2) were calculated to assess the effect sizes of the scenarios. The effect sizes were interpreted as follows: small (η2 < 0.06), medium (η2: 0.06–0.14), and large (η2 > 0.14) [40].

Linear mixed-effects models with individual clinicians specified as random effects were employed, to test in an exploratory analysis whether baseline characteristics of participating chiropractors explained variance of the peak force and rate of force application, with these SM characteristics being the main parameters investigated in the existing literature [12, 14, 41]. A full model incorporating all potentially relevant characteristics, namely age, sex, clinical experience, weight, height, BMI, grip strength, country of education, and utilized technique, was used as start. A random intercept was included for each individual clinician, allowing for variability in the baseline across different chiropractors. Subsequently, multicollinearity was addressed by iteratively eliminating correlated variables of lesser significance, followed by further removal of variables demonstrating minimal explanatory power. Model refinement was guided by Akaike information criterion (AIC) and Bayesian information criterion (BIC) metrics to assess improvement in model fit following predictor removal [42], considering the model with lower AIC/BIC superior to the one with higher values. In cases of discrepancy between the BIC and AIC, the less complex model was prioritized (the one with the lower BIC). It was ensured that the model assumptions were met by assessing diagnostic plots of residuals and fitted values.

#### Repeatability

To evaluate repeatability, mean absolute differences (MAD) between the thrusts within visits and absolute differences between visits were calculated. Further, a two-way random model intraclass correlation coefficient (ICC(2,1)) was employed, with clinicians and sessions (scenarios) treated as random effects. ICC values < 0.4 were interpreted as having poor, 0.4–0.59 fair, 0.6–0.75 good and > 0.75 excellent repeatability [43].

## Results

### Participants

Eighty-one licensed chiropractors with varying years of experience participated in the study, 32 of whom were female (39.5%). Fifty-five chiropractors (67.9%) attended both study visits. Due to a sensor malfunction, thrust data from one participant’s second visit had to be excluded. Additionally, one single thrust was excluded from analysis because the clinician reported performing the thrust for the wrong scenario. Two clinicians carried out thrusts where it was not with certainty possible for the study investigators to distinguish whether the clinicians had performed HVLA SM or mobilizations (without impulse). After visual inspection of the respective force–time profiles, it was decided to keep the data, as all typical parameters of a SM were recognizable. This led to the force–time profiles of 1240 individual thrusts being included in the analysis.

Characteristics of the participating chiropractors are summarized in Table 2. The mean age was 45.22 years (± 12.96), with females being slightly younger (40.41 years ± 13.00) compared to males (48.37 years ± 12.06). The United States (n = 33, 40,7%), Switzerland (n = 28, 34.6%), and Canada (n = 16, 19.8%) were the primary countries of education. Experience levels varied, with 59 (72.8%) clinicians having over five years and 14 (17.3%) clinicians less than three years of experience.

**Table 2:** Descriptive statistics of the participating chiropractors

Baseline characteristics	(n = 81)
Age, years ± SD	45.22 ± 12.96
Sex, n (%)	
Female	32 (39.5)
Male	49 (60.5)
Weight, kg ± SD	75.16 ± 13.65
Height, cm ± SD	174.83 ± 8.27
BMI kg/m^2^	24.47 ± 3.49
Righthanded (%)	88.9
Grip Strength right, N ± SD	47.30 ± 12.16
Grip Strength left, N ± SD	44.60 ± 11.67
Country of Education, n (multiple possible)	
Switzerland	28
Canada	16
United States	33
Great Britain	6
France	4
Experience, n	
< 3 years	14
3–5 years	8
> 5 years	59
Preferred technique in clinical practice, n	
Prone thoracic	53
Supine thoracic	26
Sitting/standing	2
Preferred hand contact in clinical practice, n	
Bilateral thenar	16
Bilateral hypothenar	10
Crossed bilateral	47
Other	8

n = number; N = Newton; SD = standard deviation; SM = spinal manipulation

Seventy-two (88.9%) of the clinicians were right handed, with a mean grip strength of 47.30 N (± 12.16) for the right hand and 44.60 N (± 11.67) for the left hand. The preferred technique for mid-thoracic SM was prone (posterior to anterior) thoracic [37] (n = 53, 65.4%) with a preference for crossed bilateral contact (n = 47, 58.0%) (see supplementary file 2 for a description of hand positions).

In their evaluation of the experimental setting, 61 (75,3%) of the participating clinicians reported that the intervention set-up was either not comparable or only somehow comparable to their clinical routine, and 71 (87.7%) that the manikin was either not comparable or only somehow comparable to a patient. The most often mentioned inadequacies were the absence of tactile feedback (n = 49, 60.5%), such as sensing muscle tension, tissue properties, and the stiffness or elasticity of the spine, and the lack of patient interaction (n = 10, 12.3%). Additionally, many clinicians (n = 16, 19.8%) found the manikin too stiff.

### Variability

Similar to their preferred contact in clinical practice, most clinicians (62.81% of thrusts) used crossed bilateral contact. This was followed by bilateral thenar (15.70%), unilateral hypothenar (9.92%), and phalangeal metacarpal (9.92%). Other techniques were rare, with bilateral hypothenar at 1.24% and thumbs at 0.41%.

The variability in forces used by different clinicians when performing an SM thrust for the same case scenario was substantial, with preload forces ranging from 4.50 to 450.25 N and peak forces between 146.08 and 1285.17 N (see Table 3). The average preload force for the 50-year-old male scenario was 146.89 ± 95.57 N, for the 70-year-old woman 126.20 ± 87.48 N, and for the 30-year-old athlete scenario 173.00 ± 105.43 N. Clinicians adjusted their peak force between each case scenario. The highest peak forces were seen in the 30-year-old athlete scenario (694.95 ± 248.24 N), compared to the 50-year-old man (615.38 ± 215.10 N) and the 70-year-old woman (406.32 ± 187.88 N). Maximum thrust speeds between 2291.67 and 18,058.33 N/s were reached, while average rate of force application ranged between 1128.07 and 9129.86 N/s.

**Table 3:** Variability of Force–time characteristics across scenarios

Force–time characteristic	Case Scenario									Statistics
	“Standard” 50 year-old male			70 year-old female			30 year-old athlete			
	Mean ± SD	Range	Interquartile range	Mean ± SD	Range	Interquartile range	Mean ± SD	Range	Interquartile range	
Preload force (N)	146.89 ± 95.57	4.50–450.25	128.83	126.20 ± 87.48	7.42–459.67	96.25	173.00 ± 105.43	5.42–398.58	146.25	(F[2,237] = 2.029 *p* = 0.134 η^2^ = 0.02 [0.00, 1.00]
Release Force (N)	112.96 ± 83.03	1.00–414.17	101.33	110.21 ± 85.48	5.83–427.58	95.58	124.30 ± 82.89	0.75–384.83	123.42	F[2,237] = 0.813 *p* = 0.445 η^2^ = 0.01 [0.00, 1.00]
Δ Preload and release force (N)	33.93 ± 33.32	0.00–112.80	49.52	15.99 ± 15.97	0.00–62.33	22.50	48.70 ± 42.78	0.00–178.33	61.92	F[2,237] = 3.948 *p* = 0.021 η^2^ = 0.03 [0.00, 1.00]
Peak force (N)	615.38 ± 215.10	146.08–1167.25	309.58	406.32 ± 187.88	89.00–912.83	235.50	694.95 ± 248.24	192.25–1285.17	376.83	F[2,237] = 11.771 *p* = < 0.0001 η^2^ = 0.09 [0.04,1.00]
Thrust force (N)	502.42 ± 184.44	144.08–1076.75	252.83	296.11 ± 143.07	40.67–805.00	170.92	570.65 ± 209.12	191.5–1129.00	321.42	F[2,237] = 14.23 *p* = < 0.0001 η^2^ = 0.11 [0.05, 1.00]
Maximum thrust speed (N/s)	7551.68 ± 2985.37	2291.67–16,733.33	4020.83	4323.15 ± 2154.11	675.00–12516.67	2537.50	8679.64 ± 3468.42	2300.00–18058.33	4883.33	(F[2,237] = 13.790 *p* = 0.0002 η^2^ = 0.10 [0.05, 1.00]
Rate of force application (N/s)	3772.48 ± 1564.83	1128.07 –7737.77	2097.37	2163.75 ± 1146.35	282.53–5966.52	1226.05	4406.50 ± 1871.46	1318.70–9129.86	2852.81	F[2,237] = 14.075 *p* = < 0.0001 η^2^ = 0.11 [0.05,1.00]
Thrust impulse (Ns)	6705.34 ± 3117.47	2000.80–16894.47	4465.68	3571.41 ± 1972.08	445.23–10,579.82	2528.37	6648.54 ± 2765.50	2274.26–13,491.53	3830.76	F[2,237] = 9.115 *p* = 0.0004 η^2^ = 0.07 [0.02, 1.00]
Preload duration (ms)	170.44 ± 121.99	8.50–687.67	117.83	149.55 ± 88.44	2.33–423.00	78.21	148.63 ± 9 4.62	4.17–486.67	90.83	F[2,237] = 0.035 *p* = 0.965 η^2^ = 0.01 [0.00, 1.00]
Thrust duration (ms)	20.38 ± 33.03	7.67–197.67	3.83	22.53 ± 33.93	7.17–275.67	7.67	20.57 ± 33.95	6.33–236.33	3.67	F[2,237] = 0.334 *p* = 0.717 η^2^ = 0.01 [0.00, 1.00]
Resolution duration (ms)	56.72 ± 28.90	6.67–150.67	32.00	56.43 ± 30.21	4.67–157.33	35.50	58.10 ± 29.83	7.33–133.33	35.17	F[2,237] = 0.274 *p* = 0.760 η^2^ = 0.01 [0.00, 1.00]
Total duration (ms)	249.82 ± 124.32	68.83–736.00	147.00	230.00 ± 107.79	62.83–601.33	136.67	229.52 ± 107.70	72.83–682.67	106.00	F[2,237] = 0.061 *p* = 0.940 η^2^ = 0.01 [0.00, 1.00]

N = Newton; N/s = Newtons per second; Ns = Newton second; ms = millisecond.

**Fig. 5:**
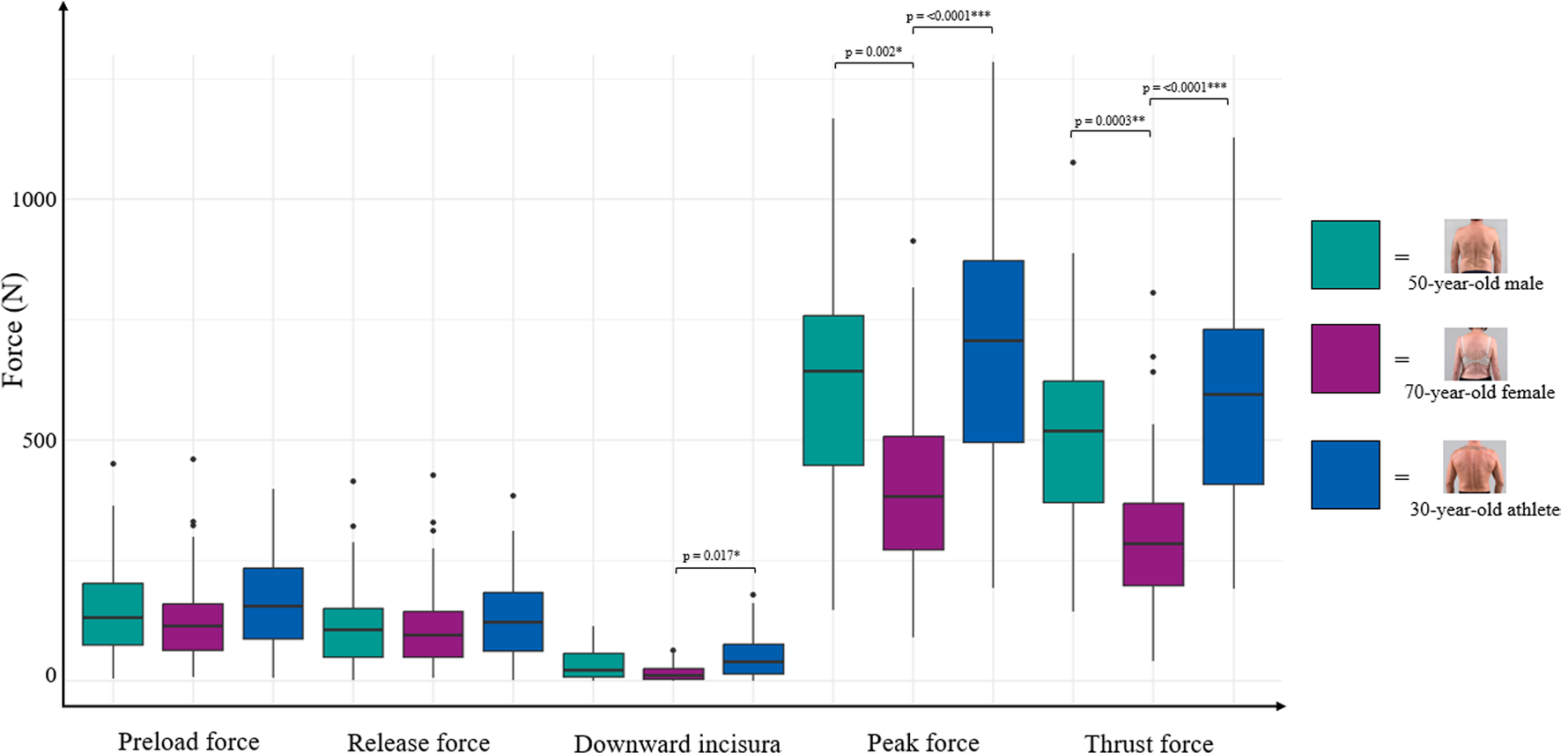
Differences in force characteristics between case scenarios. Box plots representing the distribution of forces. (Black line indicating median of the distribution, box representing the lower to upper quartile values of the data, whiskers extending to the last data point beyond 1.5 times the Interquartile Range, points representing outliers)

**Fig. 6:**
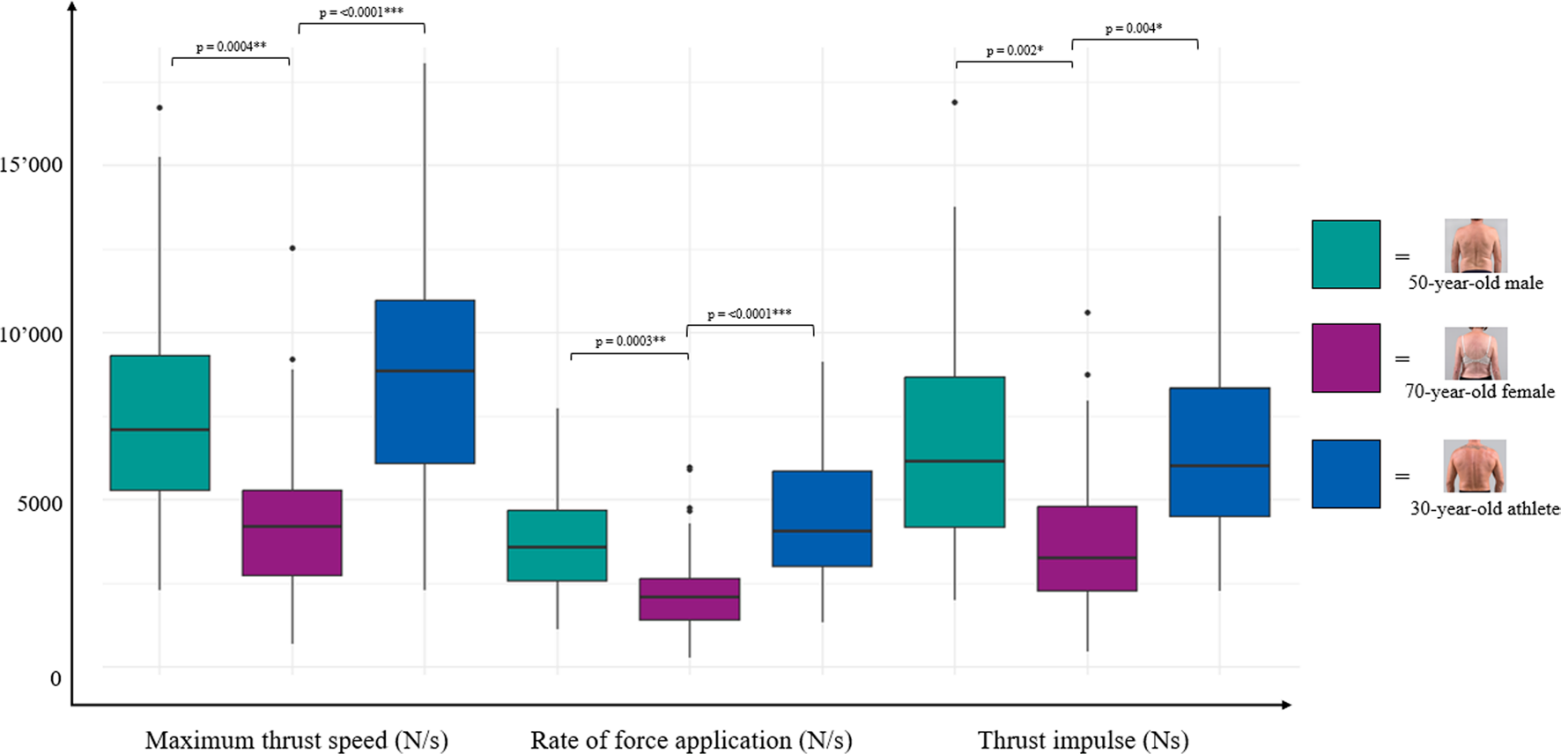
Differences in thrust speed and impulse between case scenarios. Box plots representing the distribution of forces. (Black line indicating median of the distribution, box representing the lower to upper quartile values of the data, whiskers extending to the last data point beyond 1.5 times the Interquartile Range, points representing outliers)

**Fig. 7:**
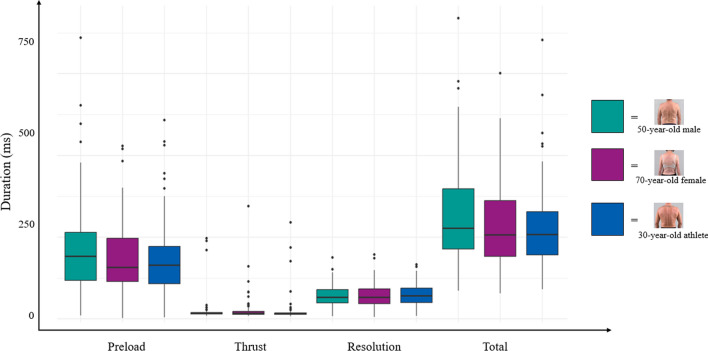
Differences in duration characteristics between case scenarios. Box plots representing the distribution of forces. (Black line indicating median of the distribution, box representing the lower to upper quartile values of the data, whiskers extending to the last data point beyond 1.5 times the Interquartile Range, points representing outliers)

A significant impact of the scenarios was seen for the differences between preload and release force (F = 3.948, *p* = 0.021, η2 = 0.03), peak force (F = 11.771, *p* =  < 0.0001, η2 = 0.09), thrust force (F = 14.233, *p* =  < 0.0001, η2 = 0.11), as well as maximum thrust speed (F = 13.790, *p* = 0.0002, η2 = 0.10), rate of force application (F = 14.075, *p* =  < 0.0001, η2 = 0.11), and thrust impulse (F = 9.115, *p* = 0.0004, η2 = 0.07), with small to medium effect sizes. The case scenarios did not have a significant impact on preload force (F = 2.029, *p* = 0.134, η2 = 0.02) and release force (F = 0.813, *p* = 0.445, η2 = 0.01). Duration metrics such as preload duration, thrust duration, resolution duration, and total duration were not different between scenarios (see Table 3 and Figs. 5, 6, 7).

**Fig. 8:**
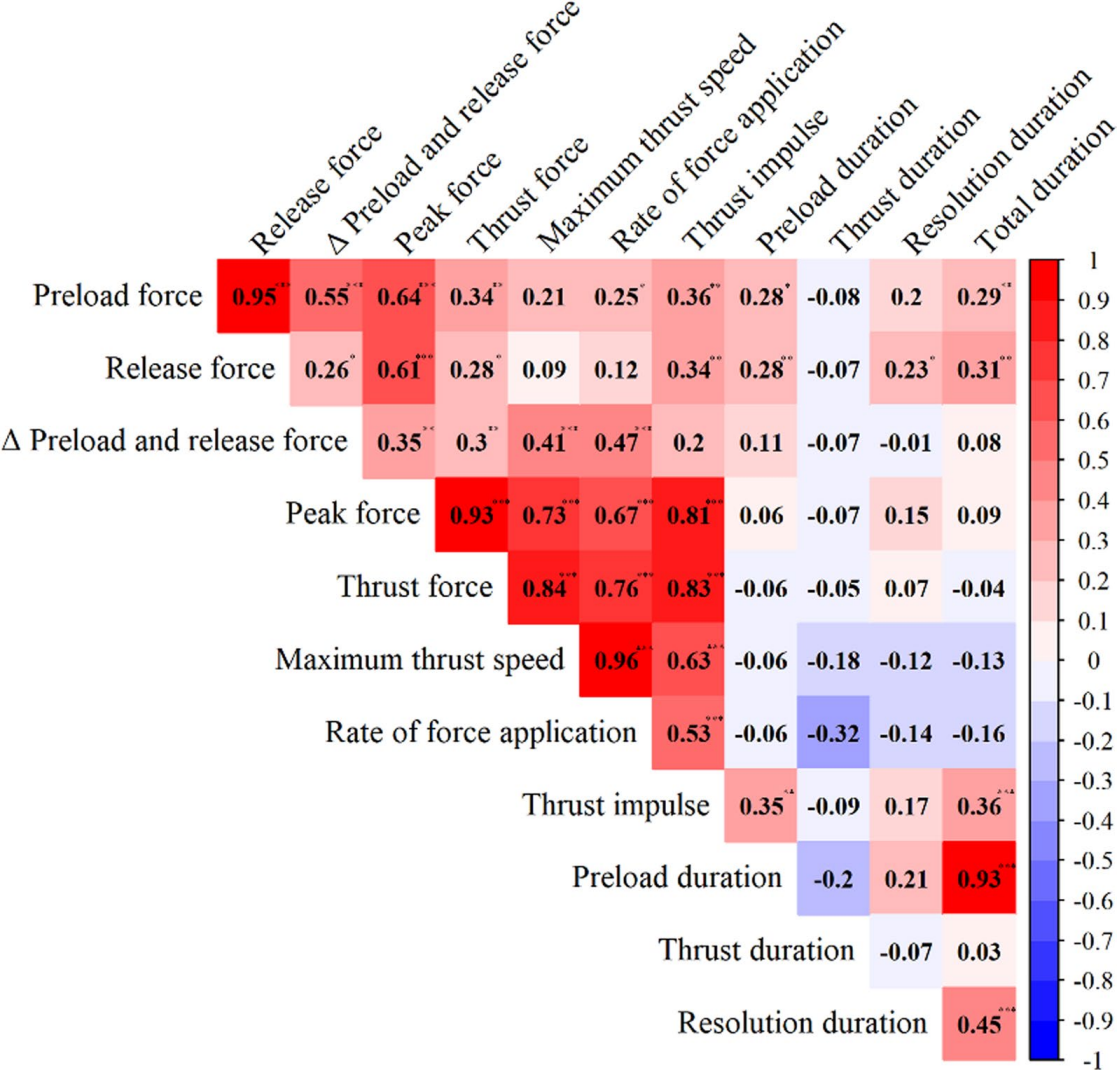
Correlation between force–time characteristics. The heatmap visualizes the correlation matrix of the analyzed characteristics. The color gradient represents the strength and direction of correlations, with blue indicating negative correlations and red indicating positive correlations. Significance is indicated by asterisks, with *p < 0.05, **p < 0.01, and ***p < 0.001

To better understand the connection between individual SM characteristics, correlations between the different characteristics of the force–time profiles were assessed (see Fig. 8). Peak force showed high correlations with maximum thrust speed (r = 0.73, *p* < 0.001), and rate of force application (r = 0.65, *p* < 0.001), indicating that higher thrust speed is needed to generate high peak forces. Preload duration and total duration were highly correlated (r = 0.93, *p* < 0.001), which can be explained by the short duration of the actual impulse in relation to the total duration of the thrust.

#### Baseline characteristics of clinicians explaining variability in peak force

Table 4 presents the results of models estimating whether clinician characteristics explain variability in peak force. For peak force, significant effects were found in the final model for grip strength (t-value = 3.567, *p*-value < 0.001), as well as certain SM techniques, namely crossed pisiform (t-value = 8.182, *p*-value < 0.001), knife edge (t-value = 2.342, *p*-value = 0.019), and unilateral hypothenar (t-value = 4.754, *p*-value < 0.001). Results of the intermediate models can be found in supplementary file 3. Comparison of the final model to the null model showed that inclusion of grip strength and used technique significantly improved the model fit (*p* < 0.0001). The marginal R^2^ indicated that the fixed effects alone explained approximately 30.5% of the variability in peak force. The conditional R^2^ suggested that both fixed and random effects combined accounted for approximately 55.6% of the variability[44].

**Table 4:** Model estimating whether baseline characteristics of the clinicians explain variability in peak force

	Estimate	SE	t-value	*p*-value
Full model. AIC = 3208.9; BIC = 3278.4				
Intercept	− 2230.070	2530.912	− 0.881	
Sex^a^	− 88.244	63.457	− 1.391	0.164
Age	− 3.414	2.254	− 1.515	0.130
Weight	− 12.82	18.046	− 0.710	0.478
Height	50.084	53.856	0.930	0.352
BMI	12.640	14.606	0.865	0.387
Grip strength (dominant hand)	6.709	2.780	2.413	0.016*
Country of education (Switzerland as reference)				
Canada	83.525	69.051	1.210	0.226
USA	75.342	64.441	1.169	0.242
Great Britain	137.451	74.582	1.843	0.065
France	110.539	91.477	1.208	0.227
Clinical experience (0–3 years as reference)				
> 5 years	− 8.054	74.296	− 0.108	0.914
3–5 years	56.151	69.296	0.810	0.418
Technique used (bilateral hypothenar as reference)				
Crossed bilateral	322.240	40.167	8.022	< 0.001*
Bilateral thenar	− 55.858	159.665	− 0.350	0.726
Knife edge	123.632	56.480	2.189	0.029
Unilateral hypothenar	321.908	60.726	5.301	< 0.001*
Thumbs	20.660	189.428	0.109	0.913
Final model. AIC = 3206.9; BIC = 3238.2				
Intercept	76.944	80.337	0.958	
Grip strength (dominant hand)	5.279	1.480	3.567	< 0.001*
Technique used (bilateral hypothenar as reference)				
Crossed bilateral	323.434	39.530	8.182	< 0.001*
Bilateral thenar	− 80.869	160.327	− 0.504	0.614
Knife edge	129.095	55.118	2.342	0.019*
Unilateral hypothenar	290.973	61.208	4.754	< 0.001*
Thumbs	1.014	188.424	0.005	0.996

SE = Standard error; *p < 0.05; AIC= Akaike information criterion; BIC = Bayesian information criterion

#### Baseline characteristics of the clinicians explaining variability in rate of force application

Table 5 presents the results of models estimating whether clinician characteristics explain variability in the rate of force application. For the rate of force application, our final model revealed significant effects for grip strength (t-value = 3.136, *p*-value = 0.002) and the use of the crossed pisiform technique (t-value = 6.033, *p*-value < 0.001). No statistically significant effects were observed for the remaining techniques. Compared to the null model, our model’s fit significantly improved with the inclusion of grip strength and the used technique (*p* < 0.0001). However, the final model’s marginal R^2^ value suggested that the fixed effects alone only accounted for approximately 20.2% of the variance, while the conditional R^2^ indicated that both fixed and random effects combined explained approximately 44.2% of the variability. Additional details on the intermediate models can be found in supplementary file 4.

**Table 5:** Model estimating whether baseline characteristics of the clinicians explain variability in rate of force application

	Estimate	SE	t-value	*p*-value
Full model. AIC = 4216.1; BIC = 4285.6				
Intercept	2665.373	20,565.98	0.130	
Sex^a^	− 330.524	515.799	− 0.641	0.522
Age	15.760	18.319	0.86	0.390
Weight	30.758	146.639	0.210	0.834
Height	− 101.959	437.641	− 0.233	0.816
BMI	− 12.380	118.688	− 0.104	0.917
Grip strength (dominant)	43.152	22.596	1.910	0.056
Country of education (Switzerland as reference)				
Canada	559.911	561.300	0.998	0.318
USA	343.159	523.850	0.655	0.512
Great Britain	1424.639	606.161	2.350	0.019
France	807.273	743.554	1.086	0.277
Clinical experience (0–3 years as reference)				
> 5 years	− 1101.38	603.800	− 1.824	0.068
3–5 years	− 251.309	563.178	− 0.446	0.656
Technique used (bilateral hypothenar as reference)				
Crossed bilateral	1900.884	329.819	5.763	< 0.001*
Bilateral thenar	− 582.514	1298.038	− 0.449	0.653
Knife edge	704.258	464.273	1.517	0.129
Unilateral hypothenar	701.659	496.853	1.412	0.158
Thumbs	122.814	1562.558	0.079	0.937
Final Model: AIC = 4204.8; BIC = 4236.1				
Intercept	430.812	619.900	0.695	
Grip strength (dominant)	35.504	11.322	3.136	0.002*
Technique used (bilateral hypothenar as reference)				
Crossed bilateral	1904.9	315.739	6.033	< 0.001*
Bilateral thenar	− 683.793	1226.878	− 0.557	0.578
Knife edge	777.124	441.972	1.758	0.079
Unilateral hypothenar	612.379	481.825	1.271	0.204
Thumbs	118.942	1538.854	0.077	0.939

SE = Standard error; *p < 0.05; AIC = Akaike information criterion; BIC = Bayesian information criterion

### Repeatability

**Fig. 9:**
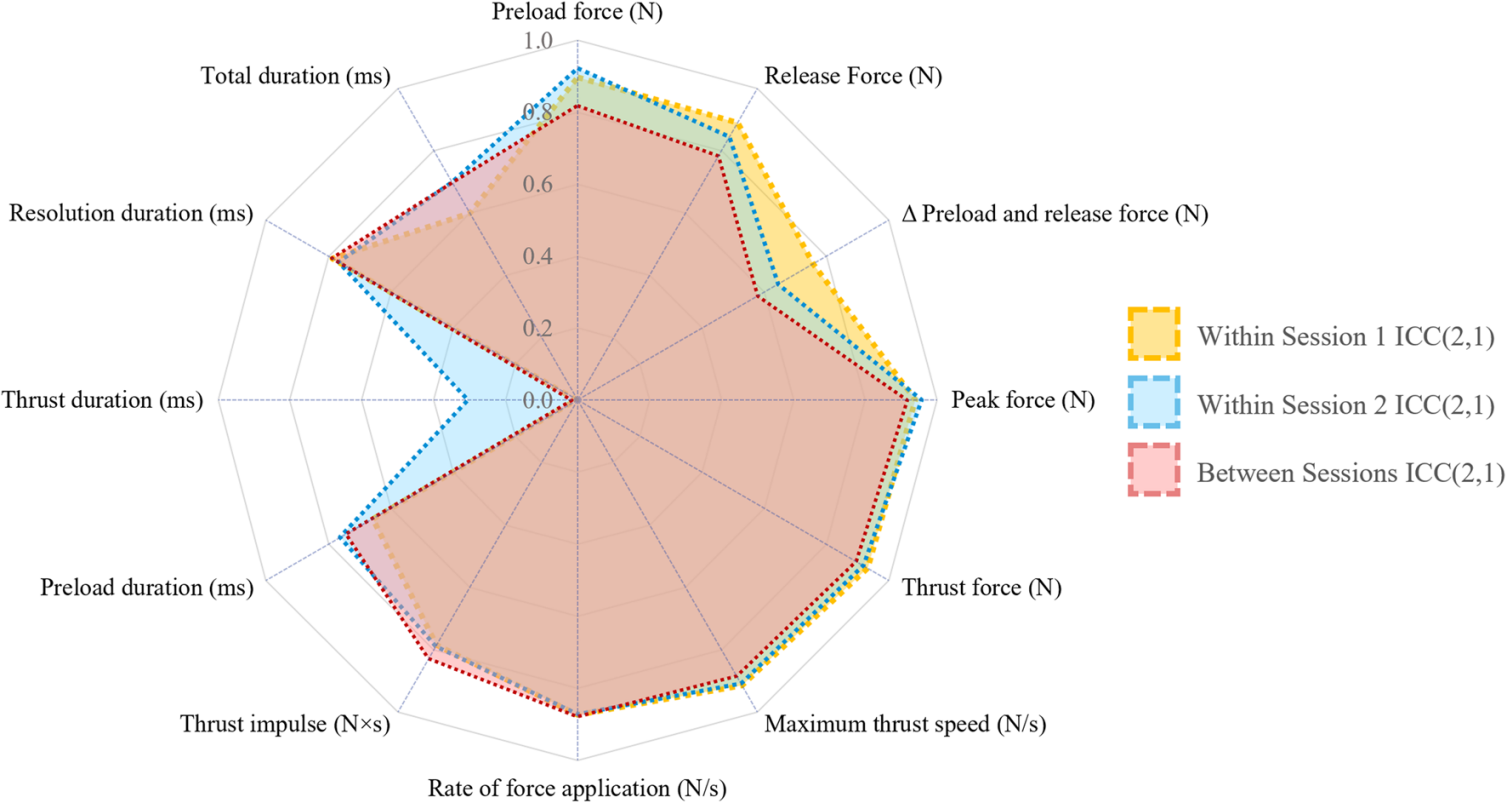
Repeatability of force–time characteristics. This radar chart illustrates the Intraclass Correlation Coefficient (ICC) values for the distinct force–time characteristics across sessions. ICC values for session 1 (yellow), session 2 (blue), and between sessions (red). Higher ICC values indicate greater repeatability and consistency

Clinicians demonstrated fair to excellent repeatability of their thrusts both within and between sessions, with all force–time characteristics remaining very consistent with ICCs values between 0.578 and 0.957. With the exception of thrust duration, also duration parameters were relatively stable across sessions, with ICC values between 0.600 and 0.789. MAD and ICC(2,1) are summarized in Table 6. As shown in Fig. 9, most force–time characteristics demonstrated high repeatability with ICC values generally above 0.8 across all sessions. However, thrust duration exhibited lower repeatability, particularly between sessions, with ICC values significantly lower than other characteristics.

**Table 6:** Repeatability of force–time characteristics within and between sessions

Characteristic	Within Session 1			Within Session 2			Between Sessions		
	MAD ± SD	ICC(2,1)	CI, *p*-value	MAD ± SD	ICC(2,1)	CI, *p*-value	MAD ± SD	ICC(2,1)	CI, *p*-value
Preload force (N)	19.52 ± 20.32	0.896	0.873–0.916, < 0.001	18.35 ± 23.42	0.922	0.901–0.940, < 0.001	34.25 ± 34.68	0.819	0.761–0.864, < 0.001
Release Force (N)	16.88 ± 17.85	0.890	0.866–0.911, < 0.001	17.41 ± 21.30	0.844	0.804–0.879, < 0.001	31.94 ± 33.45	0.783	0.716–0.836, < 0.001
Δ Preload and release force (N)	9.16 ± 13.32	0.757	0.710–0.799, < 0.001	11.32 ± 19.09	0.644	0.568–0.714, < 0.001	14.80 ± 23.95	0.578	0.466–0.672, < 0.001
Peak force (N)	39.57 ± 41.94	0.940	0.926–0.951, < 0.001	37.73 ± 33.49	0.957	0.945–0.967, < 0.001	57.06 ± 53.05	0.916	0.888–0.938, < 0.001
Thrust force (N)	37.44 ± 34.54	0.932	0.917–0.945, < 0.001	38.21 ± 36.83	0.918	0.895–0.937, < 0.001	54.06 ± 54.03	0.894	0.858–0.921, < 0.001
Maximum thrust speed (N/s)	651.24 ± 658.70	0.915	0.596–0.931, < 0.001	732.78 ± 672.22	0.909	0.884–0.930, < 0.001	941.92 ± 892.90	0.884	0.845–0.913, < 0.001
Rate of force application (N/s)	413.51 ± 450.39	0.874	0.847–0.897, < 0.001	386.71 ± 404.74	0.873	0.839–0.902, < 0.001	488.71 ± 477.70	0.879	0.838–0.909, < 0.001
Thrust impulse (Ns)	757.12 ± 773.18	0.784	0.742–0.822, < 0.001	691.08 ± 853.28	0.790	0.738–0.835, < 0.001	982.07 ± 895.78	0.828	0.773–0.871, < 0.001
Preload duration (ms)	30.39 ± 38.14	0.658	0.598–0.713, < 0.001	22.33 ± 35.15	0.762	0.704–0.812, < 0.001	37.01 ± 40.66	0.744	0.666–0.805, < 0.001
Thrust duration (ms)	1.33 ± 2.59	0.003	0.000–0.074, 0.522	2.37 ± 8.94	0.305	0.207–0.406, 0.406	7.44 ± 29.67	0.017	0.000–0.170, 0.415
Resolution duration (ms)	6.67 ± 8.27	0.787	0.745–0.825, < 0.001	6.07 ± 6.43	0.767	0.710–0.816, < 0.001	11.17 ± 10.96	0.789	0.724–0.841, < 0.001
Total duration (ms)	22.13 ± 32.71	0.600	0.534–0.662, < 0.001	25.95 ± 37.77	0.699	0.630–0.759, < 0.001	47.44 ± 48.32	0.698	0.609–0.769, < 0.001

N = Newton; N/s = Newtons per second; Ns = Newton second; ms = millisecond; MAD = mean absolute difference; SD = standard deviation; CI = 95% confidence interval

## Discussion

This study examined the variability and repeatability of thoracic SM force–time characteristics among a cohort of Swiss chiropractors. Notably, substantial variability in SM was evident across clinicians, with preload force ranging from 4.50 to 450.25 N and peak force ranging from 146.08 to 1285.17 N. While some chiropractors apply SM with higher forces, others consistently use lower thrust forces. This suggests the existence of subgroups, or rather a continuum, among clinicians that ranges from “hard SM providers” to “soft SM providers.” This variation in practice style, which has been anecdotally noted during clinical training, has so far not been studied in terms of patient outcomes or potential adverse events.

Between the different patient case scenarios, preload and release force did not differ significantly, while peak force, thrust force, maximum thrust speed, and rate of force application exhibited significant variability (all *p*-values < 0.0001). These findings suggest that while the initial application of preload force might be consistent, the subsequent execution of the thrust and resulting peak forces vary widely depending on the patient scenario, indicating the nuanced nature of SM application and likely reflecting the practitioner’s consideration of patient characteristics and clinical context, accommodating their SM thrusts to the presentation of each case [45–47].

In our exploratory analysis, consistent with findings from previous studies, the choice of SM technique significantly contributed to variability in peak force and rate of force application, which in turn were correlated with each other [48, 49]. Certain SM techniques, such as crossed bilateral, knife edge, and unilateral hypothenar were associated with higher peak force compared to other techniques. These results reflect the impact of technique selection on force modulation [49]. Additionally, clinicians with higher grip strength exhibited greater peak force and higher rate of force application during SM, indicating a potential association between muscular strength in the hand and force exertion [50]. While baseline characteristics such as sex, age, weight, height, and BMI were included in the models, their associations were inconsistent. Age showed significant associations on peak force in some intermediate models, but did not contribute significantly in the final model. Still, this suggests that demographic factors may have nuanced effects on force parameters, warranting further investigation in future studies. Interestingly, clinical experience had no influence on peak force or rate of force application. This is in contrast with previous literature which has shown that the rate of force application is related to the level of expertise [12, 14, 41]. However, while our sample consisted mainly of experienced clinicians, these previous studies addressed performance in students in a teaching environment, using a different definition of expertise. Future studies could provide valuable insights into these aspects.

Despite considerable clinician-dependent variability, thoracic SM thrusts demonstrated high repeatability, suggesting a high level of standardization in SM delivery [18]. Educational research addressing repeatability previously reported that, once a technique is learned and mastered, it can be applied consistently [51]. The only study that has investigated repeatability of SM in experienced clinicians included a small sample of 13 participants [18]. Given that SM interventions are typically tailored to individuals, research in manual medicine has not yet sufficiently addressed the description or quality assurance of SM treatments [38, 52]. Ensuring repeatability of an intervention is crucial to achieve internal validity, interpret treatment outcomes, and generalize research findings in manual therapy research[53].

### Strengths and limitations

Several limitations should be acknowledged. Firstly, while the study provided valuable insights into variability across clinicians and repeatability of force–time profiles, data collection was not performed using live human participants, limiting the generalizability of the findings to clinical settings; the use of a human analogue manikin for data collection does not fully replicate the complexities of SM delivery in clinical practice. Many clinicians reported a lack of perceptions of muscle tension, tissue properties and stiffness or elasticity of the spine during the SM interventions performed. Despite its questionable reliability, manual palpation and the identification of segmental hypomobility is a standard part of the treatment rationale and decision-making process for SM [54, 55]. Consequently, the biomechanical properties (e.g. stiffness) of the manikin and the lack of tissue properties may have directly influenced the resulting forces. However, in the context of the current literature, comparable ranges of forces were measured in studies applying SM thrusts to live human participants [22, 33]. There is currently a lack of comparative studies between the forces applied to real patients and manikins, which would be a useful addition to the current state of scientific knowledge.

Our rigorous experimental design, on the other hand, represents a significant strength and allows direct comparison of thrusts between individual clinicians. This is, to our knowledge, the largest available sample of directly comparable SM thrust records. The large number of thrusts performed in a standardized setting allows us to make transferable statements on the actual clinician-dependent variability of the SM forces applied. This variability must be recognized and considered in future studies on the efficacy and safety of manual treatments.

Additionally, the size and design of the sensors used may have influenced data collection and interpretation. Recorded forces might have been underestimated compared to the actual forces delivered due to several factors. Because of the sensor’s size, parts of the clinicians’ hands sometimes extended beyond the sensor area. However, the primary contact area of the practitioners’ hands was consistently within the sensor’s boundaries and we are confident that any ‘therapeutic’ force was captured by the sensor. This was verified through visual inspection of the two-dimensional data output from the pressure sensors. In addition, the sensors were calibrated for a peak load of 1.2 MPa, which, contrary to our expectations, was exceeded several times. However, as this was only the case in 60 out of 1240 (%) thrusts, we consider this limitation to be negligible.

## Conclusions

This is the first study to systematically investigate the variability and repeatability of SM interventions. Our findings highlight a broad range of force–time characteristics applied during thoracic SM, reflecting differences in applied techniques between involved clinicians. Despite substantial clinician-dependent variability, the high repeatability of thoracic SM thrusts suggests a level of standardization in SM delivery, indicating that chiropractors might have ‘their’ individual force–time profile that they are capable to reproduce. By further exploration of clinician-specific attributes and technique-specific factors, it may be possible to better understand variability in SM delivery in clinical practice.

Given that SM treatments are usually provided over a treatment period consisting of several consultations [56], repeatability of the intervention is an important factor for facilitating reproducible research findings. Whenever methodologically feasible, future studies on manual interventions should collect force–time characteristics, explore associations with treatment outcomes, and explore how these findings can be translated into more effective training programs to enhance the consistency, effectiveness, and safety of SM [34].

## Supplementary Information

Additional file 1.

Additional file 2.

Additional file 3.

Additional file 4.

## Data Availability

The datasets supporting the conclusions of this article are available from the corresponding author upon reasonable request.

## Abbreviations

AIC: Akaia information criterion
ANOVA: Analysis of variance
BIC: Bayesian information criterion.
CMCC: Canadian memorial chiropractic college
HVLA: High-velocity, low-amplitude
HAM™: Human-analogue manikin
HSD: Honestly significant difference
ICC: Intraclass correlation coefficient
IQR: Interquartile range
KEK: Kantonale Ethikkomission Zürich
MAD: Mean absolute difference
MPa: Megapascal
ms: Millisecond
N: Newton
Ns: Newton second
N/S: Newtons per second
SD: Standard deviation
SE: Standard error
SM: Spinal manipulation

## References

[1] Vos T, Lim SS, Abbafati C, Abbas KM, Abbasi M, Abbasifard M. Global burden of 369 diseases and injuries in 204 countries and territories, 1990–2019: a systematic analysis for the global burden of disease study 2019. Lancet. 2020. 10.1016/S0140-6736(20)30925-9. 33069326 10.1016/S0140-6736(20)30925-9 PMC7567026

[2] Ferreira ML, Luca K, Haile LM, Steinmetz JD, Culbreth GT, Cross M. Global, regional, and national burden of low back pain, 1990–2020, its attributable risk factors, and projections to 2050: a systematic analysis of the global burden of disease study 2021. Lancet Rheumatol. 2023. 10.1016/S2665-9913(23)00098-X. 10.1016/S2665-9913(23)00098-X PMC10234592 37273833

[3] Safiri S, Kolahi AA, Cross M, Hill C, Smith E, Carson-Chahhoud K. Prevalence, deaths, and disability-adjusted life years due to musculoskeletal disorders for 195 countries and territories 1990–2017. Arthritis Rheumatol. 2021. 10.1002/art.41571. 33150702 10.1002/art.41571

[4] Briggs AM, Smith AJ, Straker LM, Bragge P. Thoracic spine pain in the general population: prevalence, incidence and associated factors in children, adolescents and adults, A systematic review. BMC Musculoskelet Disord. 2009;29(10):77. 10.1186/1471-2474-10-77 PMC2720379 19563667

[5] Paige NM, Miake-Lye IM, Booth MS, Beroes JM, Mardian AS, Dougherty P, et al. Association of spinal manipulative therapy with clinical benefit and harm for acute low back pain: systematic review and meta-analysis. JAMA. 2017;317(14):1451–60. 28399251 10.1001/jama.2017.3086 PMC5470352

[6] Gross A, Langevin P, Burnie SJ, Bedard-Brochu MS, Empey B, Dugas E, et al. Manipulation and mobilisation for neck pain contrasted against an inactive control or another active treatment. Cochrane Database Syst Rev. 2015;9:Cd04249. 10.1002/14651858.CD004249.pub4 PMC10883412 26397370

[7] Hurwitz EL, Goldstein MS, Morgenstern H, Chiang LM. The impact of psychosocial factors on neck pain and disability outcomes among primary care patients: results from the UCLA neck pain study. Disabil Rehabil. 2006;28(21):1319–29. 17083180 10.1080/09638280600641509

[8] Rubinstein SM, van Middelkoop M, Assendelft WJJ, de Boer MR, van Tulder MW. Spinal manipulative therapy for chronic low-back pain: an update of a Cochrane review. Spine. 2011;36(13):E825-846. 21593658 10.1097/BRS.0b013e3182197fe1

[9] Corp N, Mansell G, Stynes S, Wynne-Jones G, Morsø L, Hill JC, et al. Evidence-based treatment recommendations for neck and low back pain across Europe: a systematic review of guidelines. Eur J Pain Lond Engl. 2021;25(2):275–95. 10.1002/ejp.1679 PMC7839780 33064878

[10] Oliveira CB, Maher CG, Pinto RZ, Traeger AC, Lin CWC, Chenot JF, et al. Clinical practice guidelines for the management of non-specific low back pain in primary care: an updated overview. Eur Spine J Off Publ Eur Spine Soc Eur Spinal Deform Soc Eur Sect Cerv Spine Res Soc. 2018;27(11):2791–803. 10.1007/s00586-018-5673-2 29971708

[11] Descarreaux M, Dugas C, Lalanne K, Vincelette M, Normand MC. Learning spinal manipulation: the importance of augmented feedback relating to various kinetic parameters. Spine J Off J North Am Spine Soc. 2006;6(2):138–45. 10.1016/j.spinee.2005.07.001 16517384

[12] Triano JJ, Gissler T, Forgie M, Milwid D. Maturation in rate of high-velocity, low-amplitude force development. J Manipulative Physiol Ther. 2011;34(3):173–80. 21492752 10.1016/j.jmpt.2011.02.007

[13] Herzog W. The biomechanics of spinal manipulation. J Bodyw Mov Ther. 2010. 10.1016/j.jbmt.2010.03.004. 20538226 10.1016/j.jbmt.2010.03.004

[14] Descarreaux M, Dugas C. Learning spinal manipulation skills: assessment of biomechanical parameters in a 5-year longitudinal study. J Manipulative Physiol Ther. 2010;33(3):226–30. 20350677 10.1016/j.jmpt.2010.01.011

[15] Gevers-Montoro C, Provencher B, Descarreaux M, Ortega de Mues A, Piché M. Neurophysiological mechanisms of chiropractic spinal manipulation for spine pain. Eur J Pain Lond Engl. 2021;25(7):1429–48. 10.1002/ejp.1773 33786932

[16] Vigotsky AD, Bruhns RP. The role of descending modulation in manual therapy and its analgesic implications: a narrative review. Pain Res Treat. 2015;2015: 292805. 26788367 10.1155/2015/292805 PMC4695672

[17] Bialosky JE, Beneciuk JM, Bishop MD, Coronado RA, Penza CW, Simon CB, et al. Unraveling the mechanisms of manual therapy: modeling an approach. J Orthop Sports Phys Ther. 2018;48(1):8–18. 29034802 10.2519/jospt.2018.7476

[18] Triano JJ, Giuliano D, Kanga I, Starmer D, Brazeau J, Screaton CE, et al. Consistency and malleability of manipulation performance in experienced clinicians: a pre-post experimental design. J Manipulat Physiol Ther. 2015;38(6):407–15. 10.1016/j.jmpt.2015.05.002 26198595

[19] Triano JJ, Rogers CM, Combs S, Potts D, Sorrels K. Developing skilled performance of lumbar spine manipulation. J Manipulat Physiol Ther. 2002;25(6):353–61. 10.1067/mmt.2002.126132 12183693

[20] Downie AS, Vemulpad S, Bull PW. Quantifying the high-velocity, low-amplitude spinal manipulative thrust: a systematic review. J Manipulat Physiol Ther. 2010;33(7):542–53. 10.1016/j.jmpt.2010.08.001 20937432

[21] Gyer G, Michael J, Inklebarger J, Ibne AI. Effects of biomechanical parameters of spinal manipulation: a critical literature review. J Integr Med. 2022;20(1):4–12. 34756673 10.1016/j.joim.2021.10.002

[22] Gorrell LM, Nyirö L, Pasquier M, Pagé I, Heneghan NR, Schweinhardt P, et al. Spinal manipulation characteristics: a scoping literature review of force-time characteristics. Chiropr Man Ther. 2023;31(1):36. 10.1186/s12998-023-00512-1 PMC10500795 37705030

[23] Gorrell LM, Conway PJ, Herzog W. Differences in force-time parameters and electromyographic characteristics of two high-velocity, low-amplitude spinal manipulations following one another in quick succession. Chiropr Man Ther. 2020;8(28):67. 10.1186/s12998-020-00355-0 PMC7722317 33287851

[24] Haldeman S. Neurological effects of the adjustment. J Manipulat Physiol Ther. 2000;23(2):112–4. 10.1016/s0161-4754(00)90078-2 10714538

[25] Pasquier M, Daneau C, Marchand AA, Lardon A, Descarreaux M. Spinal manipulation frequency and dosage effects on clinical and physiological outcomes: a scoping review. Chiropr Man Ther. 2019;27:23. 10.1186/s12998-019-0244-0 PMC6530068 31139346

[26] Reed WR, Cao DY, Long CR, Kawchuk GN, Pickar JG. Relationship between biomechanical characteristics of spinal manipulation and neural responses in an animal model: effect of linear control of thrust displacement versus force, thrust amplitude, thrust duration, and thrust rate. Evid Based Complement Altern Med. 2013;2013:492039. 10.1155/2013/492039 PMC3563165 23401713

[27] Pickar JG, Sung PS, Kang YM, Ge W. Response of lumbar paraspinal muscles spindles is greater to spinal manipulative loading compared with slower loading under length control. Spine J Off J North Am Spine Soc. 2007;7(5):583–95. 10.1016/j.spinee.2006.10.006 PMC2075482 17905321

[28] Cao DY, Reed WR, Long CR, Kawchuk GN, Pickar JG. Effects of thrust amplitude and duration of high-velocity, low-amplitude spinal manipulation on lumbar muscle spindle responses to vertebral position and movement. J Manipulat Physiol Ther. 2013;36(2):68–77. 10.1016/j.jmpt.2013.01.004 PMC3752031 23499141

[29] Nougarou F, Dugas C, Loranger M, Pagé I, Descarreaux M. The role of preload forces in spinal manipulation: experimental investigation of kinematic and electromyographic responses in healthy adults. J Manipulat Physiol Ther. 2014;37(5):287–93. 10.1016/j.jmpt.2014.04.002 24928637

[30] Pagé I, Nougarou F, Dugas C, Descarreaux M. The effect of spinal manipulation impulse duration on spine neuromechanical responses. J Can Chiropr Assoc. 2014;58(2):141–8. 24932018 PMC4025084

[31] Nougarou F, Dugas C, Deslauriers C, Pagé I, Descarreaux M. Physiological responses to spinal manipulation therapy: investigation of the relationship between electromyographic responses and peak force. J Manipulat Physiol Ther. 2013;36(9):557–63. 10.1016/j.jmpt.2013.08.006 24161387

[32] Pasquier M, Young JJ, Lardon A, Descarreaux M. Factors associated with clinical responses to spinal manipulation in patients with non-specific thoracic back pain: a prospective cohort study. Front Pain Res. 2022;6(2): 742119. 10.3389/fpain.2021.742119 PMC8915706 35295527

[33] Choi G, Giuliano D, Tibbles A, Howarth SJ, Tran S, Lee J, et al. Investigating force-time characteristics of prone thoracic SMT and self-reported patient outcome measures: a feasibility study. Chiropr Man Ther. 2023;31(1):19. 10.1186/s12998-023-00491-3 PMC10329299 37420257

[34] Kirchhoff KT, Dille CA. Issues in intervention research: maintaining integrity. Appl Nurs Res ANR. 1994;7(1):32–8. 8203878 10.1016/0897-1897(94)90018-3

[35] Herzog W, Conway PJ, Kawchuk GN, Zhang Y, Hasler EM. Forces exerted during spinal manipulative therapy. Spine. 1993;18(9):1206–12. 8362328 10.1097/00007632-199307000-00014

[36] Forand D, Drover J, Suleman Z, Symons B, Herzog W. The forces applied by female and male chiropractors during thoracic spinal manipulation. J Manipulative Physiol Ther. 2004;27(1):49–56. 14739874 10.1016/j.jmpt.2003.11.006

[37] Bergmann TF, Peterson DH. Chiropractic technique: principles and procedures. 3rd ed. Churchill Livingstone. Elselvier; 2011.

[38] Groeneweg R, Rubinstein SM, Oostendorp RAB, Ostelo RWJG, Tulder MW. Guideline for reporting interventions on spinal manipulative therapy: consensus on interventions reporting criteria list for spinal manipulative therapy (CIRCLe SMT). J Manip Physiol Ther. 2017. 10.1016/j.jmpt.2016.10.013. 10.1016/j.jmpt.2016.10.013 28017603

[39] Elgendi M, Cecchini V, Nyirö L, Gorrell LM, Schweinhardt P, Menon C. Analysis of force profile features in spinal manipulation therapy. IEEE Access. 2023;11:133386–93.

[40] Wilkinson L. Statistical methods in psychology journals: Guidelines and explanations. Am Psychol. 1999;54(8):594–604.

[41] Cohen E, Triano JJ, McGregor M, Papakyriakou M. Biomechanical performance of spinal manipulation therapy by newly trained vs. practicing providers: does experience transfer to unfamiliar procedures? J Manipulative Physiol Ther. 1995;18(6):347–52. 7595108

[42] Wang X, Andrinopoulou ER, Veen KM, Bogers AJJC, Takkenberg JJM. Statistical primer: an introduction to the application of linear mixed-effects models in cardiothoracic surgery outcomes research—a case study using homograft pulmonary valve replacement data. Eur J Cardio-Thorac Surg Off J Eur Assoc Cardio-Thorac Surg. 2022;62(4):ezac429. 10.1093/ejcts/ezac429 PMC9496250 36005884

[43] Shrout PE, Fleiss JL. Intraclass correlations: uses in assessing rater reliability. Psychol Bull. 1979;86(2):420–8. 18839484 10.1037//0033-2909.86.2.420

[44] Nakagawa S, Schielzeth H. A general and simple method for obtaining R2 from generalized linear mixed-effects models. Methods Ecol Evol. 2013;4(2):133–42.

[45] Funabashi M, Son J, Pecora CG, Tran S, Lee J, Howarth SJ, et al. Characterization of thoracic spinal manipulation and mobilization forces in older adults. Clin Biomech Bristol Avon. 2021;89: 105450. 34450432 10.1016/j.clinbiomech.2021.105450

[46] Triano JJ, McGregor M, Skogsbergh DR. Use of chiropractic manipulation in lumbar rehabilitation. J Rehabil Res Dev. 1997;34(4):394–404. 9323643

[47] Hawk C, Schneider MJ, Haas M, Katz P, Dougherty P, Gleberzon B, et al. Best practices for chiropractic care for older adults: a systematic review and consensus update. J Manipulative Physiol Ther. 2017;40(4):217–29. 28302309 10.1016/j.jmpt.2017.02.001

[48] Joo S, Kim J, Lee Y, Song C. The biomechanical analysis of magnitude and direction of force by different techniques of thoracic spinal manipulation. BioMed Res Int. 2020;26(2020): e8928071. 10.1155/2020/8928071 PMC7399734 32775447

[49] Cambridge EDJ, Triano JJ, Ross JK, Abbott MS. Comparison of force development strategies of spinal manipulation used for thoracic pain. Man Ther. 2012;17(3):241–5. 22386279 10.1016/j.math.2012.02.003

[50] Vaishya R, Misra A, Vaish A, Ursino N, D’Ambrosi R. Hand grip strength as a proposed new vital sign of health: a narrative review of evidences. J Health Popul Nutr. 2024;9(43):7. 10.1186/s41043-024-00500-y PMC10777545 38195493

[51] Triano JJ, Descarreaux M, Dugas C. Biomechanics–review of approaches for performance training in spinal manipulation. J Electromyogr Kinesiol Off J Int Soc Electrophysiol Kinesiol. 2012;22(5):732–9. 10.1016/j.jelekin.2012.03.011 22542770

[52] Glasziou P, Meats E, Heneghan C, Shepperd S. What is missing from descriptions of treatment in trials and reviews? BMJ. 2008;336(7659):1472–4. 18583680 10.1136/bmj.39590.732037.47 PMC2440840

[53] De Vet H. TC Mokkink L, Knol D. Measurement in Medicine. New York: Cambridge University Press; 2011. 348 p. (Practical Guides to Biostatistics and Epidemiology; vol. 1).

[54] Nyberg RE, Russell SA. The science of spinal motion palpation: a review and update with implications for assessment and intervention. J Man Manip Ther. 2013;21(3):160–7. 24421627 10.1179/2042618613Y.0000000029 PMC3744849

[55] Hicks GE, Fritz JM, Delitto A, Mishock J. Interrater reliability of clinical examination measures for identification of lumbar segmental instability. Arch Phys Med Rehabil. 2003;84(12):1858–64. 14669195 10.1016/s0003-9993(03)00365-4

[56] Shekelle PG, Adams AH, Chassin MR, Hurwitz EL, Brook RH. Spinal manipulation for low-back pain. Ann Intern Med. 1992;117(7):590–8. 1388006 10.7326/0003-4819-117-7-590

