# Supplementary material for: Variability and repeatability of spinal manipulation force–time characteristics in thoracic spinal manipulation on a manikin
Source: Chiropr Man Therap. 2024 Nov 11;32:33. doi: 10.1186/s12998-024-00551-2 (PMC11552221; doi:10.1186/s12998-024-00551-2)
Supplement: Supplementary file 1 — Additional file 1. [file 12998_2024_551_MOESM1_ESM.pdf]

# "Standard" male patient

Please provide a posterior-anterior mid-thoracic spinal manipulative thrust (SM). Position your hands as you usually would in clinical practice:

Imagine the following scenario:

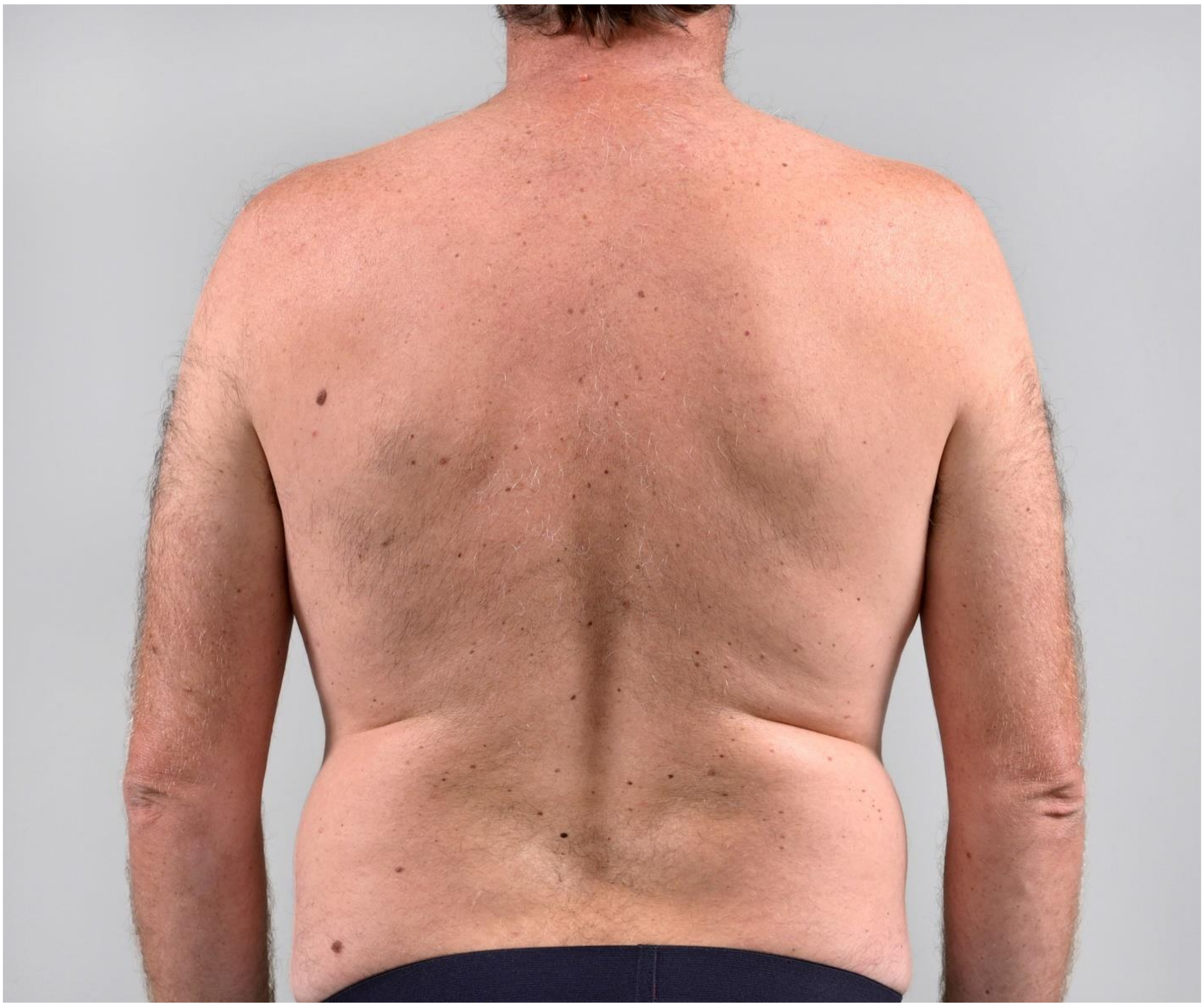

- 50-year-old healthy male patient
- 180 cm, normal body weight for height
- returning visit for sporadic musculoskeletal mid-thoracic pain
- no radiation and no signs or symptoms of inflammatory or degenerative pathologies
- previously responding well to mid-thoracic SM

# Young male athlete

Please provide a posterior-anterior mid-thoracic spinal manipulative thrust (SM). Position your hands as you usually would in clinical practice:

Imagine the following scenario:

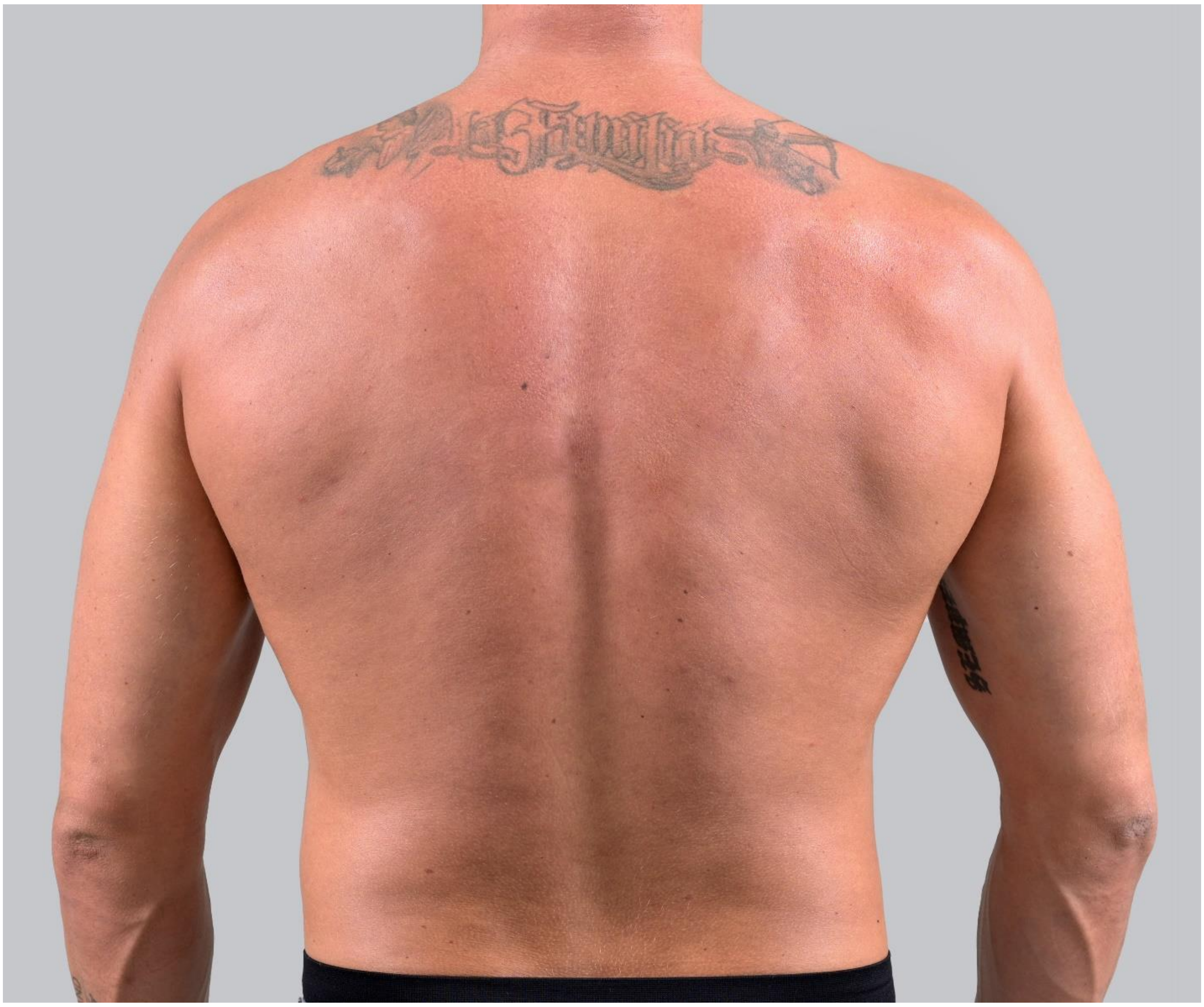

- 30-year-old healthy male patient
- 180 cm, very athletic body type
- returning visit for sporadic musculoskeletal mid-thoracic pain
- no radiation and no signs or symptoms of inflammatory or degenerative pathologies
- previously responding well to mid-thoracic SM

# Elderly female patient

Please provide a posterior-anterior mid-thoracic spinal manipulative thrust (SM). Position your hands as you usually would in clinical practice:

Imagine the following scenario:

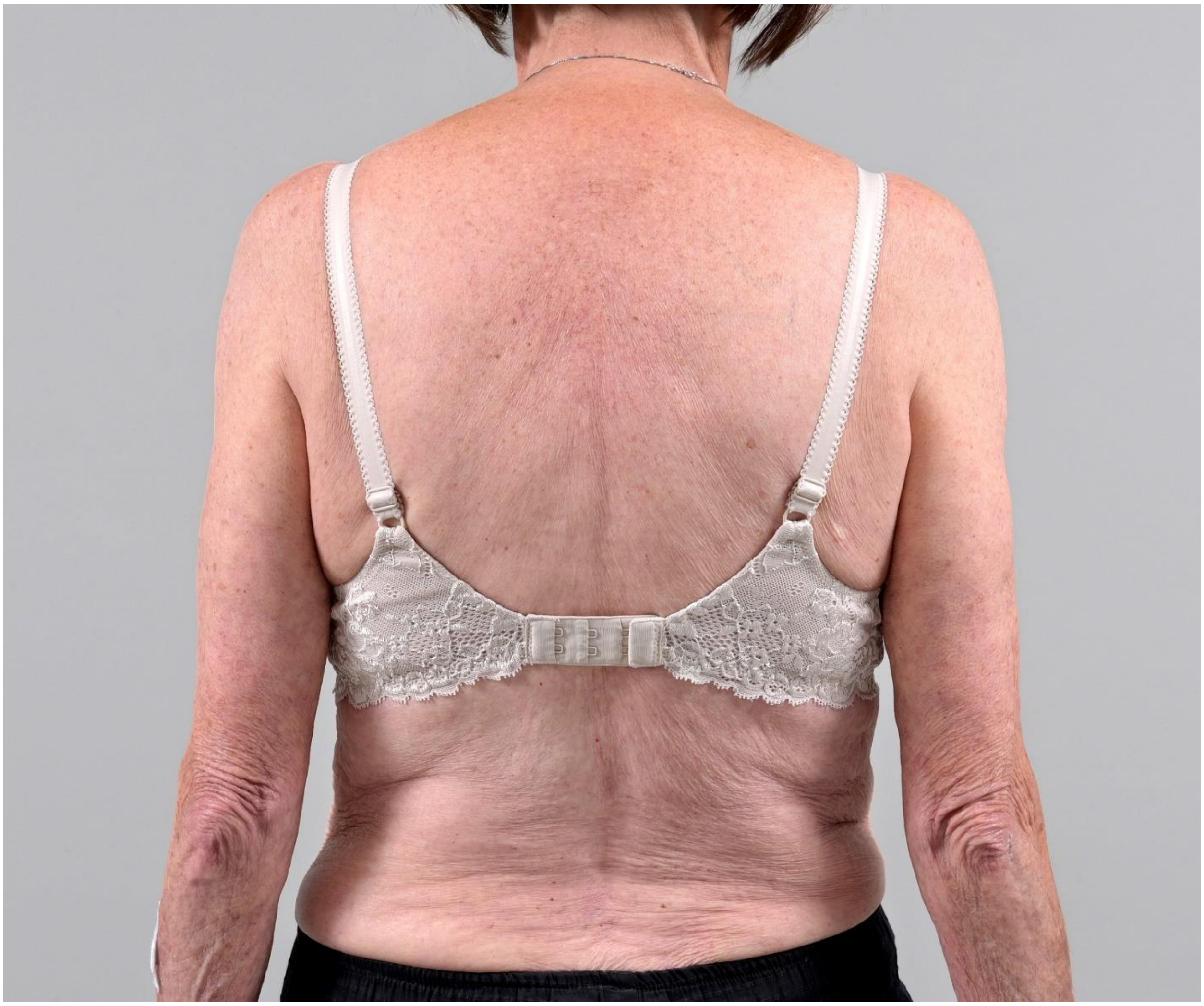

- 70-year-old healthy female patient
- 170 cm, normal body weight for height
- returning visit for sporadic musculoskeletal mid-thoracic pain
- no radiation and no signs or symptoms of inflammatory conditions
- moderate degeneration of the thoracic spine, slightly decreased bone density
- previously responding well to mid-thoracic SM
