# Supplementary material for: Variability and repeatability of spinal manipulation force–time characteristics in thoracic spinal manipulation on a manikin
Source: Chiropr Man Therap. 2024 Nov 11;32:33. doi: 10.1186/s12998-024-00551-2 (PMC11552221; doi:10.1186/s12998-024-00551-2)

Hand positions

Bilateral thenar

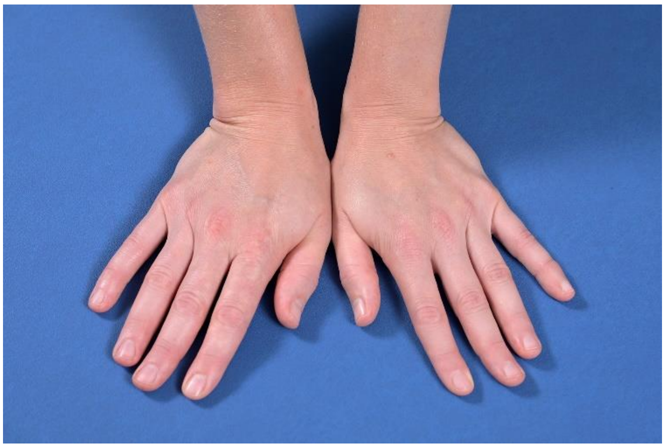

Bilateral hypothenar

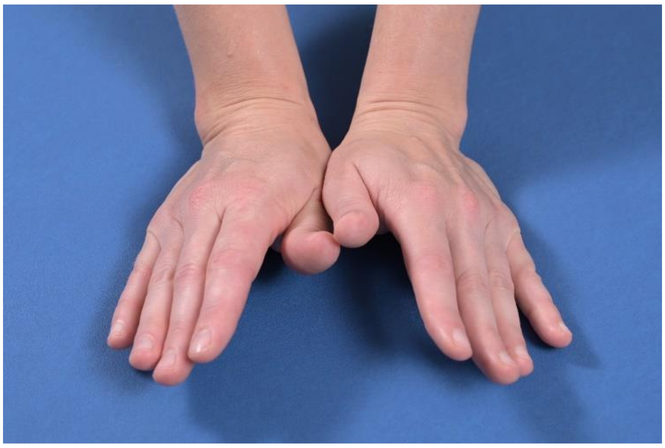

Unilateral hypothenar (reinforced)

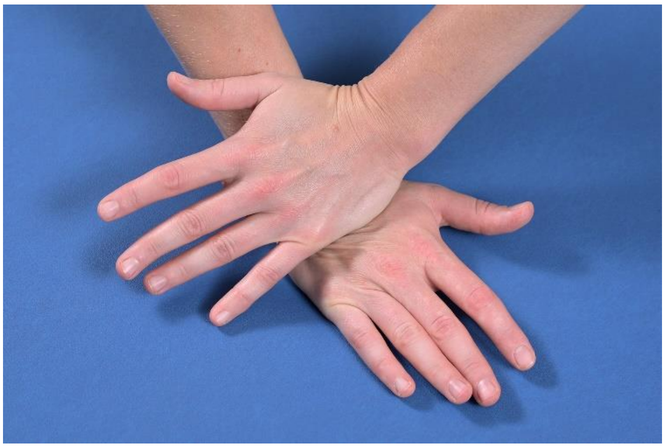

Crossed bilateral (torque)

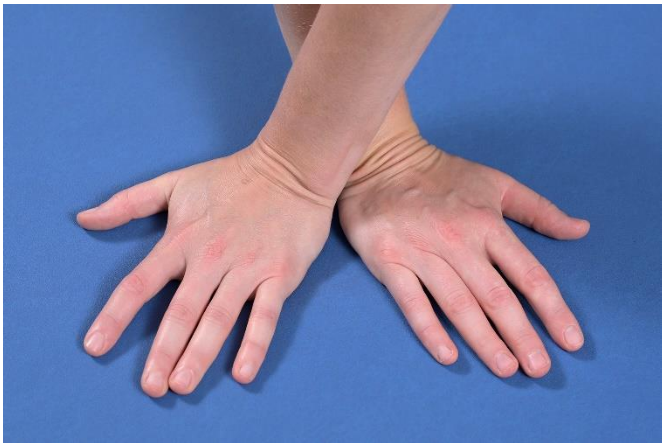

Thumbs

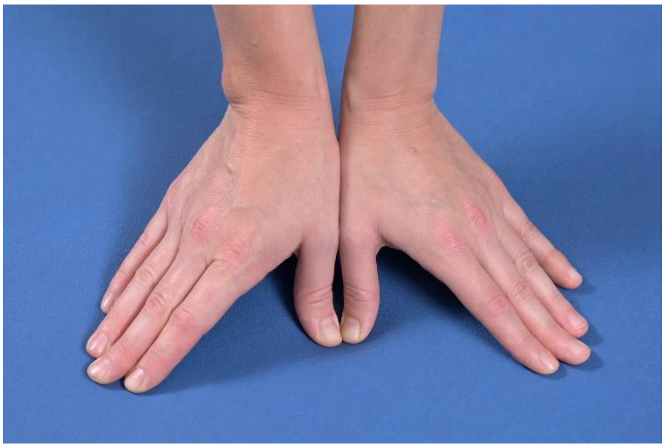

Phalangeal metacarpal (knife edge)

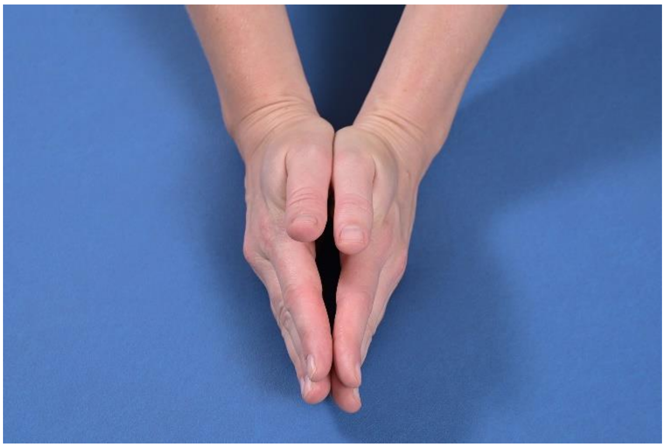

Supplement: Supplementary file 2 — Additional file 2. [file 12998_2024_551_MOESM2_ESM.pdf]
