# Supplementary material for: Variability and repeatability of spinal manipulation force–time characteristics in thoracic spinal manipulation on a manikin
Source: Chiropr Man Therap. 2024 Nov 11;32:33. doi: 10.1186/s12998-024-00551-2 (PMC11552221; doi:10.1186/s12998-024-00551-2)
Supplement: Supplementary file 3 — Additional file 3. [file 12998_2024_551_MOESM3_ESM.pdf]

Supplementary file 3. Nested models for peak force

	Estimate	SE	t-value	p-value
**Full model. AIC = 3208.9; BIC = 3278.4**				
Intercept	-2230.070	2530.912	-0.881	
Sex[a]	-88.244	63.457	-1.391	0.164
Age	-3.414	2.254	-1.515	0.130
Weight	-12.82	18.046	-0.710	0.478
Height	50.084	53.856	0.930	0.352
BMI	12.640	14.606	0.865	0.387
**Grip strength (dominant)**	**6.709**	**2.780**	**2.413**	**0.016***
Country of education				
Canada	83.525	69.051	1.210	0.226
USA	75.342	64.441	1.169	0.242
Great Britain	137.451	74.582	1.843	0.065
France	110.539	91.477	1.208	0.227
Clinical experience				
>5 years	-8.054	74.296	-0.108	0.914
3-5 years	56.151	69.296	0.810	0.418
Technique used				
Crossed bilateral	**322.240**	**40.167**	**8.022**	**< 0.001***
Bilateral thenar	-55.858	159.665	-0.350	0.726
Knife edge	123.632	56.480	2.189	0.029
Unilateral hypothenar	**321.908**	**60.726**	**5.301**	**< 0.001***
Thumbs	20.660	189.428	0.109	0.913
**Model 2: Correlated characteristics removed. AIC = 3208.0; BIC = 3263.6**				
Intercept	-113.239	509.700	-0.222	
Sex[a]	-87.627	60.292	-1.453	0.146
**Age**	**-4.044**	**1.880**	**-2.151**	**0.031***
Height	1.913	3.398	0.563	0.573
**Grip strength (dominant)**	**7.272**	**2.505**	**2.902**	**0.004***
Country of education				
Canada	89.074	61.687	1.444	0.149
USA	100.712	56.088	1.796	0.072
Great Britain	130.821	71.098	1.84	0.066
France	121.001	82.951	1.459	0.145
Technique used				
Crossed bilateral	**332.084**	**40.024**	**8.297**	**< 0.001***
Bilateral thenar	-0.877	158.301	-0.006	0.995
Knife edge	**148.719**	**55.686**	**2.671**	**0.008***
Unilateral hypothenar	**321.538**	**60.969**	**5.274**	**< 0.001***
Thumbs	26.825	189.247	0.142	0.887

	Estimate	SE	t-value	p-value
**Model 3: Height removed. AIC = 3206.3; BIC = 3258.5**				
Intercept	167.600	100.977	1.660	
Sex[a]	-78.828	57.94	-1.361	0.174
**Age**	**-3.941**	**1.862**	**-2.117**	**0.034***
**Grip strength (dominant)**	**7.927**	**2.210**	**3.587**	**< 0.001***
Country of education				
Canada	93.619	60.89	1.538	0.124
USA	107.751	54.461	1.978	0.048
Great Britain	136.836	69.958	1.956	0.050
France	119.481	82.500	1.448	0.148
Technique used				
Crossed bilateral	**332.565**	**39.941**	**8.326**	**< 0.001***
Bilateral thenar	-10.161	156.625	-0.065	0.948
Knife edge	**146.305**	**55.355**	**2.643**	**0.008***
Unilateral hypothenar	**319.934**	**60.677**	**5.273**	**< 0.001***
Thumbs	25.878	189.062	0.137	0.891
**Model 4: Sex removed. AIC = 3206.3; BIC = 3255.1**				
Intercept	182.318	101.09	1.804	
**Age**	**-4.6**	**1.811**	**-2.541**	**0.011***
**Grip strength (dominant)**	**5.641**	**1.447**	**3.897**	**< 0.001***
Country of education				
Canada	99.17	61.169	1.621	0.105
USA	106.281	54.834	1.938	0.053
Great Britain	136.909	70.454	1.943	0.052
France	113.479	82.97	1.368	0.171
Technique used				
Crossed bilateral	**331.211**	**40.047**	**8.271**	**< 0.001***
Bilateral thenar	-13.304	157.707	-0.084	0.933
Knife edge	**139.39**	**55.294**	**2.521**	**0.012***
Unilateral hypothenar	**308.882**	**60.448**	**5.11**	**< 0.001***
Thumbs	8.083	188.808	0.043	0.966
**Model 5: Country of Education removed. AIC = 3205.1; BIC = 3239.9**				
Intercept	188.457	99.165	1.9	
Age	-2.594	1.373	-1.89	0.059
**Grip strength (dominant)**	**5.492**	**1.463**	**3.753**	**< 0.001***
Technique used				
Crossed bilateral	**315.374**	**39.55**	**7.974**	**< 0.001***
Bilateral thenar	-35.392	159.792	-0.221	0.825
Knife edge	**129.368**	**54.769**	**2.362**	**0.018***
Unilateral hypothenar	**293.95**	**60.639**	**4.848**	**< 0.001***
Thumbs	-26.842	188.419	-0.142	0.887

	Estimate	SE	t-value	p-value
**Final model: Age removed. AIC = 3206.9; BIC = 3238.2**				
Intercept	76.944	80.337	0.958	
**Grip strength (dominant)**	**5.279**	**1.480**	**3.567**	**< 0.001***
Technique used				
Crossed bilateral	**323.434**	**39.530**	**8.182**	**< 0.001***
Bilateral thenar	-80.869	160.327	-0.504	0.614
Knife edge	**129.095**	**55.118**	**2.342**	**0.019***
Unilateral hypothenar	**290.973**	**61.208**	**4.754**	**< 0.001***
Thumbs	1.014	188.424	0.005	0.996

SE = Standard error; * = $p < 0.05$; AIC= Akaike information criterion; BIC = Bayesian information criterion.
