# Supplementary material for: Variability and repeatability of spinal manipulation force–time characteristics in thoracic spinal manipulation on a manikin
Source: Chiropr Man Therap. 2024 Nov 11;32:33. doi: 10.1186/s12998-024-00551-2 (PMC11552221; doi:10.1186/s12998-024-00551-2)
Supplement: Supplementary file 4 — Additional file 4. [file 12998_2024_551_MOESM4_ESM.pdf]

Supplementary file 4. Nested models for rate of force application

	Estimate	SE	t-value	p-value
**Full model. AIC = 4216.1; BIC = 4285.6**				
Intercept	2665.373	20565.98	0.130	
Sex[a]	-330.524	515.799	-0.641	0.522
Age	15.760	18.319	0.86	0.390
Weight	30.758	146.639	0.210	0.834
Height	-101.959	437.641	-0.233	0.816
BMI	-12.380	118.688	-0.104	0.917
Grip strength (dominant)	43.152	22.596	1.910	0.056
Country of education				
Canada	559.911	561.300	0.998	0.318
USA	343.159	523.850	0.655	0.512
Great Britain	1424.639	606.161	2.350	0.019
France	807.273	743.554	1.086	0.277
Clinical experience				
>5 years	-1101.38	603.800	-1.824	0.068
3-5 years	-251.309	563.178	-0.446	0.656
Technique used				
Crossed bilateral	**1900.884**	**329.819**	**5.763**	**< 0.001***
Bilateral thenar	-582.514	1298.038	-0.449	0.653
Knife edge	704.258	464.273	1.517	0.129
Unilateral hypothenar	701.659	496.853	1.412	0.158
Thumbs	122.814	1562.558	0.079	0.937
**Model 2: Correlated characteristics removed. AIC = 4212.2; BIC = 4274.8**				
Intercept	-1824.470	4077.297	-0.447	
Sex[a]	-320.066	501.891	-0.638	0.523
Age	15.987	18.044	0.886	0.376
Height	12.263	26.968	0.455	0.649
**Grip strength (dominant)**	43.095	20.417	2.111	0.035
Country of education				
Canada	550.969	552.331	0.998	0.318
USA	319.953	504.389	0.634	0.526
Great Britain	**1432.918**	**596.656**	**2.402**	**0.016***
France	803.853	710.908	1.131	0.258
Clinical experience	-			
>5 years	-1114.850	592.254	-1.882	0.060
3-5 years	-256.355	553.032	-0.464	0.643
Technique used				
Crossed pisiforme	**1896.454**	**325.358**	**5.829**	**< 0.001***
Double thenar	-618.207	1258.739	-0.491	0.623
Knife	693.768	454.159	1.528	0.127
Reinforced hypothenar	695.473	491.691	1.414	0.157
Reinforced thumb	71.458	1556.644	0.046	0.963

	Estimate	SE	t-value	p-value
**Model 3: Height removed. AIC = 4210.5; BIC = 4269.6**				
Intercept	-15.872	874.838	-0.018	
Sex[a]	-259.431	481.299	-0.539	0.590
Age	16.511	17.900	0.922	0.357
**Grip strength (dominant)**	**47.161**	**18.247**	**2.585**	**0.010***
Country of education				
Canada	577.806	546.077	1.058	0.290
USA	363.044	492.726	0.737	0.461
Great Britain	**1469.874**	**587.539**	**2.502**	**0.012***
France	789.878	706.095	1.119	0.263
Clinical experience				
>5 years	-1114.22	588.793	-1.892	0.058
3-5 years	-269.649	549.146	-0.491	0.623
Technique used				
Crossed pisiforme	**1898.914**	**324.509**	**5.852**	**< 0.001***
Double thenar	-677.544	1244.357	-0.544	0.586
Knife	679.737	451.347	1.506	0.132
Reinforced hypothenar	681.497	488.897	1.394	0.163
Reinforced thumb	45.379	1554.699	0.029	0.977
**Model 4: Sex removed. AIC = 4208.8; BIC = 4264.4**				
Intercept	79.169	851.52	0.093	
Age	13.379	16.838	0.795	0.427
**Grip strength (dominant)**	**39.512**	**11.373**	**3.474**	**0.001***
Country of education				
Canada	573.255	542.833	1.056	0.291
USA	339.703	487.893	0.696	0.486
Great Britain	**1455.002**	**583.483**	**2.494**	**0.013**
France	741.213	696.107	1.065	0.287
Clinical experience				
>5 years	-1085.720	582.916	-1.863	0.062
3-5 years	-340.508	530.438	-0.642	0.521
Technique used				
Crossed pisiforme	1898.141	323.654	5.865	**< 0.001***
Double thenar	-674.924	1237.149	-0.546	0.585
Knife	670.465	449.653	1.491	0.136
Reinforced hypothenar	645.110	482.436	1.337	0.181
Reinforced thumb	-37.312	1548.087	-0.024	0.981

	Estimate	SE	t-value	p-value
**Model 5: Age removed. AIC = 4207.5; BIC = 4259.7**				
Intercept	467.527	694.234	0.673	
**Grip strength (dominant)**	**39.536**	**11.337**	**3.487**	**< 0.001***
Country of education				
Canada	640.394	534.561	1.198	0.231
USA	420.364	475.775	0.884	0.377
Great Britain	**1434.285**	**581.055**	**2.468**	**0.014***
France	676.057	689.028	0.981	0.327
Clinical experience				
>5 years	-851.048	500.768	-1.699	0.089
3-5 years	-331.523	528.614	-0.627	0.531
Technique used				
Crossed pisiforme	**1895.332**	**323.189**	**5.864**	**< 0.001***
Double thenar	-510.247	1216.026	-0.42	0.674
Knife	704.028	447.42	1.574	0.115
Reinforced hypothenar	657.741	481.224	1.367	0.172
Reinforced thumb	-55.484	1547.41	-0.036	0.971
**Model 6: Clinical experience removed. AIC = 4206.8; BIC = 4252.0**				
Intercept	319.889	672.416	0.476	
**Grip strength (dominant)**	**37.587**	**11.348**	**3.312**	**0.001**
Country of education				
Canada	53.973	400.062	0.135	0.893
USA	-142.374	335.501	-0.424	0.672
Great Britain	**1079.658**	**545.504**	**1.979**	**0.048***
France	296.882	648.587	0.458	0.647
Technique used				
Crossed bilateral	**1857.954**	**323.585**	**5.742**	**< 0.001***
Bilateral thenar	-544.390	1225.505	-0.444	0.657
Knife edge	683.275	446.688	1.530	0.126
Unilateral hypothenar	614.982	482.850	1.274	0.203
Thumbs	85.375	1545.42	0.055	0.956
**Final Model: Country of education removed. AIC = 4204.8; BIC = 4236.1**				
Intercept	430.812	619.900	0.695	
**Grip strength (dominant)**	**35.504**	**11.322**	**3.136**	**0.002***
Technique used				
Crossed bilateral	**1904.9**	**315.739**	**6.033**	**< 0.001***
Bilateral thenar	-683.793	1226.878	-0.557	0.578
Knife edge	777.124	441.972	1.758	0.079
Unilateral hypothenar	612.379	481.825	1.271	0.204
Thumbs	118.942	1538.854	0.077	0.939

SE = Standard error; * = p < 0.05; AIC= Akaike information criterion; BIC = Bayesian information criterion.
